# Presbylarynx Revisited: A Multidimensional Approach to Laryngeal Aging and Clinical Decision-Making

**DOI:** 10.3390/diagnostics16213695

**Published:** 2026-11-09

**Authors:** Engin Dursun, Emine Elif Altuntaş, İlter Denizoğlu, Güleser Saylam, Celil Göçer, Haldun Oğuz

**Affiliations:** 1 Department of Otorhinolaryngology, Faculty of Medicine, Lokman Hekim University, 06510 Ankara, Türkiye; 2 Department of Speech Language Pathology, Faculty of Health Sciences, İzmir Tınaztepe University, 35400 İzmir, Türkiye

**Keywords:** presbylarynx, presbyphonia, aging voice, vocal fold atrophy, glottic insufficiency, multidimensional assessment, voice therapy, injection augmentation, geriatric laryngology

## Abstract

**Background:** Presbylarynx describes the structural phenotype of an aging larynx, whereas presbyphonia refers to its clinically relevant perceptual and functional expression. Although vocal fold atrophy, bowing, and glottic insufficiency are common in older adults, their presence and severity do not consistently correspond to patient-reported voice impairments. Therefore, presbylaryngeal morphology should be interpreted within the broader context of compensatory capacity, physiological reserve, systemic health, and individual communication demands. **Objective:** This narrative review aims to provide a clinically oriented and multidimensional synthesis of presbylarynx, encompassing its terminology, morphological and functional characteristics, relationship with systemic aging, differential diagnosis, clinical assessment, management strategies, and emerging diagnostic and therapeutic approaches. **Methods:** A targeted literature search of PubMed/MEDLINE, Scopus, and Web of Science was conducted in August 2026, with additional targeted searches and citation checking during revision. Relevant systematic and narrative reviews, clinical practice guidelines, consensus statements, prospective and retrospective clinical studies, translational investigations, and landmark publications addressing the presbylarynx, presbyphonia, vocal fold atrophy, laryngeal aging, assessment, and treatment were reviewed and synthesized narratively. No formal systematic review protocol, duplicate screening, standardized data extraction, or risk-of-bias assessment was performed. **Results:** Presbylarynx encompasses age-related alterations in the vocal fold epithelium, lamina propria, intrinsic laryngeal musculature, and neuromuscular control, which may produce vocal fold atrophy, bowing, glottic insufficiency, and reduced vibratory efficiency. However, structural findings alone neither establish the cause of dysphonia nor determine the need for treatment. Clinical expression is modified by respiratory function, frailty, comorbidities, cognition, hearing status, compensatory behavior, and communication requirements. Assessment should integrate laryngeal morphology with patient-reported, auditory-perceptual, acoustic, aerodynamic, respiratory, and systemic findings, while excluding alternative and coexisting causes of dysphonia. Management should target meaningful functional impairment and may include observation, behavioral voice therapy, injection augmentation, durable medialization, or combined approaches within a flexible, nonlinear, and patient-centered pathway. Artificial intelligence, digital voice biomarkers, regenerative interventions, advanced biomaterials, and neuromuscular stimulation represent emerging opportunities; however, their clinical application remains limited by insufficient validation and predominantly preliminary or preclinical evidence. **Conclusions:** The presbylarynx is not inherently pathological or an indication for treatment. Its clinical significance emerges when age-related laryngeal changes contribute to meaningful limitations in voice function, communication, or quality of life. A multidimensional, system-aware, and function-driven approach is essential for accurate interpretation and individualized management. Future research should prioritize standardized definitions, reproducible phenotyping, comparable outcome measures, prospective comparative studies, and careful validation of new technologies.

## 1. Introduction

Voice is a fundamental component of human communication and social interaction, and its integrity is closely linked to the quality of life. With the global increase in life expectancy, age-related voice disorders have emerged as a growing clinical and public health concern. Population-based and systematic studies indicate that dysphonia affects a substantial proportion of older adults, with reported prevalence rates ranging from approximately 5% to nearly 30%, depending on the study population and case definition [1,2,3]. Despite this high prevalence, voice disorders in older adults remain underdiagnosed and undertreated and are frequently perceived as inevitable consequences of “normal aging” [1,4]. This misconception contributes to delayed referral and suboptimal care, even though voice impairment in older adults is associated with reduced social participation, functional decline, and diminished quality of life [1,4,5].

Within this context, presbylarynx (also termed presbylaryngis) and presbyphonia are related but distinct. Presbylarynx denotes the morphological and structural manifestations of laryngeal aging, including thinning and bowing of the vocal folds, prominence of the vocal processes, and age-related alterations of the lamina propria and intrinsic laryngeal musculature [5,6,7]. Presbyphonia, in contrast, refers to the perceptual and functional manifestations of the aging voice, typically characterized by breathiness, reduced vocal intensity, vocal fatigue, instability, and decreased endurance [8,9]. Although presbylaryngeal changes may constitute an anatomical substrate for presbyphonia, the two terms are not synonymous, and the relationship between laryngeal structure and voice function is neither linear nor deterministic [5,6,10].

Age-related laryngeal changes occur within the broader context of systemic aging. Structural alterations of the larynx develop alongside age-related changes in respiratory function, neuromuscular control, endocrine regulation, cognitive status, sensory feedback mechanisms, and overall functional capacity [5,6,8,11]. Increasing evidence suggests that presbylaryngeal findings may also be associated with the general health status of older adults, including comorbidity burden and functional dependence. Patients with endoscopic signs of the presbylarynx have been reported to exhibit a higher prevalence of chronic conditions, including diabetes mellitus and chronic respiratory disease. These observations suggest that presbylarynx may reflect biological rather than chronological aging and support interpreting laryngeal findings within a broader systemic and geriatric context rather than as an isolated local phenomenon [12].

Importantly, not all older adults with morphological signs of laryngeal aging experience clinically significant voice complaints, demonstrating that anatomical findings alone are insufficient to predict functional impairment [5,6,13]. Accordingly, presbylarynx should not be treated as a stand-alone etiological diagnosis based solely on endoscopic appearance. When dysphonia is present, attribution to presbylaryngeal change requires assessment of alternative or coexisting causes, including benign vocal fold lesions, inflammatory conditions, vocal fold paresis or paralysis, neurodegenerative disorders, and functional voice disorders [13,14]. Presbylaryngeal findings should be interpreted alongside symptom burden, functional voice assessment, compensatory capacity, systemic health, and individual communication demands.

Despite the growing body of literature on the aging voice, most reviews and original studies remain predominantly presbyphonia-centered, focusing primarily on perceptual voice characteristics and acoustic measures [8,15]. Recent narrative and scoping reviews have provided valuable syntheses of the mechanisms underlying presbyphonia [5,8,13]. However, in this literature, presbylarynx is often treated as a secondary or implicit concept rather than a distinct morphological phenotype with specific diagnostic and interpretative challenges. This relative underrepresentation limits our understanding of how presbylaryngeal findings should be interpreted in relation to systemic health, functional aging, and clinically meaningful voice impairment [5,12].

Although the histological and endoscopic manifestations of the aging larynx are well documented, fewer studies have examined how these findings should inform comprehensive clinical assessment, guide treatment selection, or predict therapeutic outcomes in older adults [5,7,11]. The central clinical challenge is therefore not merely to recognize age-related laryngeal morphology but to determine when and how these findings become functionally relevant and should influence management.

**Figure 1:**
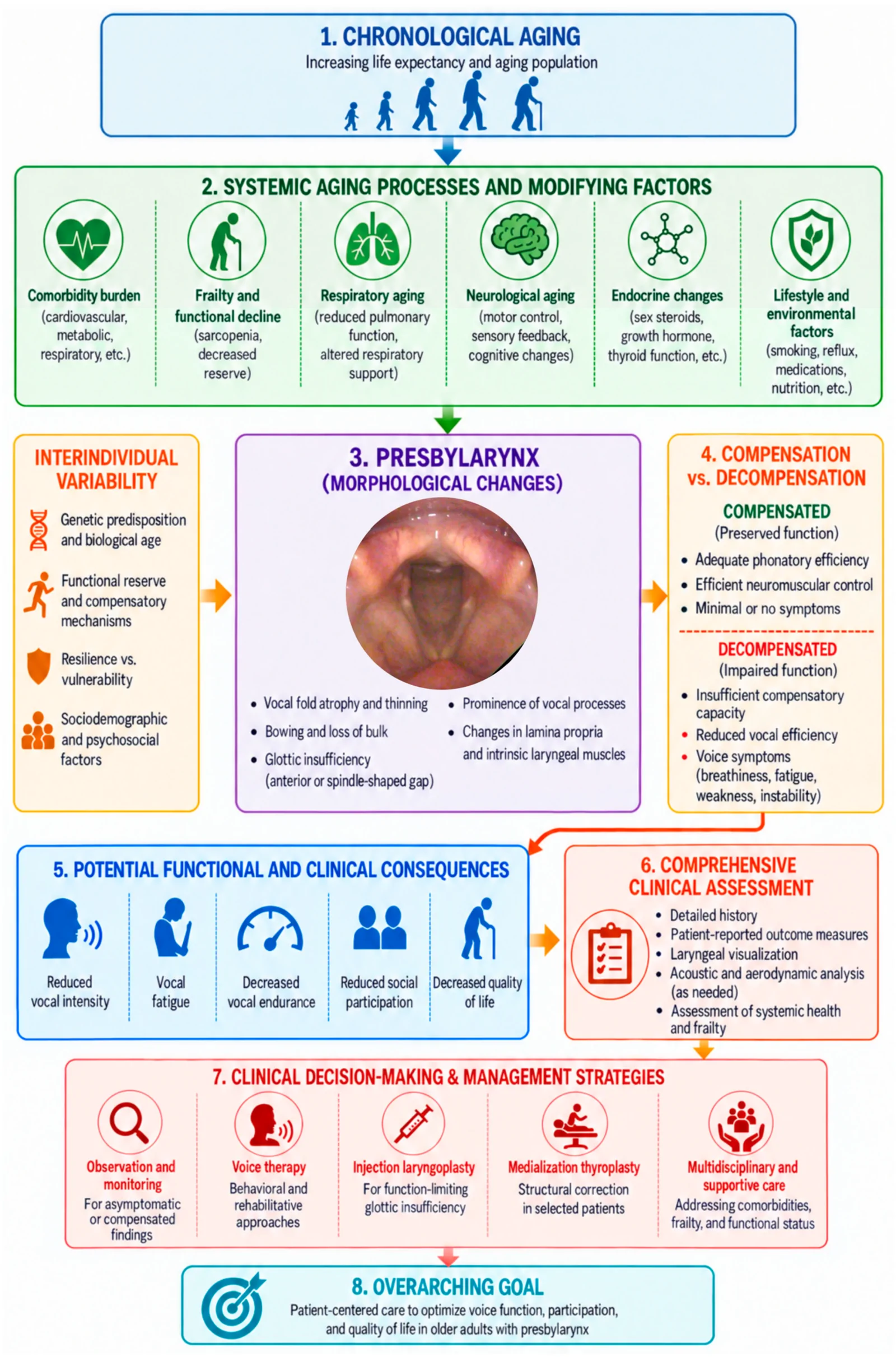
Conceptual framework of presbylarynx and individualized clinical decision-making. Original schematic created by the authors, synthesizing evidence on laryngeal morphology, systemic health, voice impairment, and multidimensional assessment [12,16,17,18,19]. The model illustrates how physiological reserve and compensatory capacity may modify clinical expression and management needs. “Compensated” and “decompensated” describe proposed functional states rather than validated diagnostic categories.

Accordingly, this review revisits the presbylarynx by integrating morphological, functional, and systemic perspectives to support clinical decision-making in older adults. Specifically, we aim to: (i) summarize the current evidence on the morphological and functional features of the presbylarynx; (ii) examine its interaction with systemic aging and comorbid conditions; (iii) highlight key considerations in differential diagnosis and clinical interpretation; and (iv) discuss the implications for patient-centered management and future research. Figure 1 summarizes the proposed conceptual model linking structural laryngeal aging, systemic factors, functional consequences, and clinical decision-making in the presbylarynx.

## 2. Literature Search and Scope of the Review

This narrative review was developed through a targeted literature search of PubMed/MEDLINE, Scopus, and Web of Science, which was completed in August 2026. Additional relevant publications were identified through targeted searches and citation checks during the revision. The search terms included combinations of “presbylarynx,” “presbyphonia,” “aging voice,” “vocal fold atrophy,” “vocal fold bowing,” “laryngeal aging,” “geriatric voice disorders,” “glottic insufficiency,” “voice therapy,” and “presbyphonia treatment.” Additional relevant studies were identified through a manual review of the reference lists of key articles and contemporary reviews.

Priority was given to systematic reviews, clinical practice guidelines, consensus statements, prospective clinical studies, relevant translational research, and landmark investigations addressing the structural, functional, and systemic aspects of laryngeal aging. Particular emphasis was placed on studies examining the distinction between the presbylarynx and presbyphonia, the relationship between presbylaryngeal morphology and voice function, the interaction between laryngeal and systemic aging, differential diagnosis, multidimensional clinical assessment, and contemporary management strategies.

The evidence was reviewed and interpreted narratively rather than through a formal systematic review or meta-analysis. No formal review protocol was registered, and we did not conduct duplicate study screening, standardized data extraction, or formal risk-of-bias assessment. The thematic synthesis, including current and emerging management approaches, was intended to support evidence-informed clinical decision-making in older adults with presbylaryngeal findings and/or voice-related complaints.

## 3. Definition and Conceptual Framework

This section traces the historical evolution of the terminology, clarifies the relationship between the presbylarynx and presbyphonia, and examines the role and limitations of the current endoscopic classification systems.

### 3.1. Terminology and Historical Background

The term presbylarynx is derived from the Greek word presbys, meaning old, and was introduced to describe the structural and morphological changes occurring in the larynx with aging. Early descriptions of the aging voice focused predominantly on perceptual characteristics, with limited distinction between the anatomical alterations of the larynx and their functional consequences [9]. Advances in endoscopic visualization, histological analysis, and objective voice assessment have allowed structural laryngeal aging to be examined separately from age-related changes in voice function.

Historically, presbylarynx has encompassed macroscopic and microscopic laryngeal alterations [7,11]. These findings were initially regarded as largely inevitable manifestations of senescence and were frequently conflated with presbyphonia. Subsequent clinical and endoscopic observations, however, showed that laryngoscopic evidence of presbylaryngeal change does not invariably result in clinically meaningful voice impairment [6,13].

### 3.2. Presbylarynx Versus Presbyphonia

Table 1 summarizes the principal conceptual and clinical distinctions between presbylarynx and presbyphonia.

Preserved respiratory support, neuromuscular reserve, and adaptive phonatory strategies may limit the functional impact of presbylaryngeal changes [6,10]. Conversely, relatively subtle morphological abnormalities may be associated with substantial voice impairment in individuals with reduced physiological reserve, frailty, respiratory limitation, neuromuscular dysfunction, or coexisting systemic disease [2,13]. Individual communication demands may further influence whether a structural change is clinically relevant.

Attribution of voice impairment to presbyphonia requires assessment of alternative and coexisting causes [8,14].

### 3.3. Endoscopic Classifications of Presbylarynx

Several classification approaches have been proposed to standardize the description and stratification of presbylaryngeal findings and to facilitate communication among clinicians and researchers. The three-type classification proposed by Santos et al. provides one such framework. In this scheme, Type 0 indicates no significant morphological alterations in the glottal configuration; it does not exclude histological or neuromuscular changes that are not visible endoscopically. Type 1 is defined by the presence of at least two presbylaryngeal findings, including vocal fold atrophy, bowing, prominence of the vocal processes, supraglottic hyperactivity, decreased vibratory amplitude, and/or vocal tremor. Type 2 comprises the features of Type 1 accompanied by glottic insufficiency, typically manifested as an anterior or spindle-shaped glottal gap [21,22].

The 2023 validation study assessed 30 rigid laryngostroboscopic recordings rated by 20 otorhinolaryngologists from five tertiary hospitals in two sessions, two weeks apart. Raters were blinded to clinical information and received an educational session before each assessment. Average intra-rater Cohen’s kappa was 0.847, while average inter-rater kappa was 0.670 and 0.691 at the two assessments [21]. These findings support reproducibility under standardized, training-supported conditions. However, observer agreement does not establish diagnostic accuracy, longitudinal progression, or prediction of treatment benefit.

This classification provides a shared vocabulary for describing the heterogeneity of presbylaryngeal morphology in older adults. However, its categories should be interpreted as morphological strata rather than linear measures of disease progression, functional severity, or treatment requirements. A higher morphological category may be associated with greater functional impairment at the group level; however, these associations vary across cohorts and do not establish an individual severity scale [23,24,25,26]. Therefore, the classification cannot independently determine whether a patient’s dysphonia is caused by presbylaryngeal changes.

In a single-center cross-sectional study of 306 older adults, Santos et al. reported higher Voice Handicap Index-10 and voice-disorder screening scores, greater perceptual impairment, and shorter maximum phonation time in Type 2 than in Types 0 and 1. Nevertheless, 23% of participants with presbyphonia were classified as Type 0, while some participants without presbyphonia had Type 1 or 2 findings [23]. Clinical applicability is limited by the small Type 2 subgroup (*n* = 23), exclusion of 21 patients with discordant endoscopic ratings, and the absence of adjustment for age, sex, or systemic and functional status. However, the authors suggested that Type 2 might represent advanced laryngeal aging and discussed type-based treatment options; neither progression nor treatment response was tested [21,23]. In another cohort of 172 adults, Türe et al. found higher shimmer and Acoustic Voice Quality Index values and lower smoothed cepstral peak prominence in Type 2 than in Type 1, without significant differences in Voice Handicap Index-10 scores across types [26]. These acoustic associations require cautious interpretation because multiple comparisons were performed and cases without observer consensus were excluded.

Other studies emphasize the incomplete correspondence between endoscopic findings and voice impairment. In 107 Cantonese-speaking older adults, Gao et al. found only small associations between presbylarynx status and selected voice measures; none remained significant after correction for multiple comparisons, and subgroup sizes were markedly unequal [25]. Crawley et al. similarly found that videostroboscopic signs of presbylarynx and laryngeal asymmetry did not distinguish 100 older adults with voice complaints from 100 without complaints [24]. These studies provide complementary evidence about structure–function relationships, rather than direct validation of all three Santos categories.

Morphological type may nevertheless contribute to treatment discussions. In a retrospective presbyphonia cohort, Haddad et al. found an association between Santos type and observed treatment choice: Type 1 patients more often received voice therapy, whereas Type 2 patients more often received injections. Patient-rated voice severity and perceptual grade also contributed to the clinical assessment [27]. Because treatment decisions already incorporated anatomical findings and patient preferences, this association does not establish that type independently predicts treatment success or that all Type 2 patients require augmentation. The study examined treatment selection rather than comparative therapeutic effectiveness.

Existing endoscopic classifications also have methodological and clinical limitations. Assessment of bowing, atrophy, glottic configuration, supraglottic activity, and vibratory amplitude remains partly subjective and may be influenced by examiner experience, image quality, examination technique, phonatory task, and patient cooperation [6,10]. The categories also combine structural findings, vibratory characteristics, and compensatory behaviors; consequently, patients within the same type may have different mechanisms of impairment. Some findings are not specific to aging and may overlap with vocal fold paresis, neurogenic disorders, benign vocal fold lesions, inflammatory disease, or compensatory muscle tension patterns [14,15]. In addition, an endoscopic classification cannot adequately capture the physiological reserve, systemic health, communication demands, or the patient’s experience of voice impairment.

Endoscopic classification should also not be treated as a proxy for body composition. In a cross-sectional study of 167 older adults, body roundness index did not differ overall between those with and without presbylarynx; lower values were observed only in the prespecified symptomatic subgroup [28]. This indirect measure of central adiposity neither quantifies muscle mass nor validates a morphological severity category.

Therefore, current presbylarynx classifications should be regarded as descriptive components of a broader multidimensional assessment rather than as stand-alone diagnostic instruments.

## 4. Morphological Changes in the Aging Larynx

The presbylarynx encompasses macroscopic, microstructural, myogenic, and neuromuscular manifestations of laryngeal aging. The rate, distribution, and extent of these changes vary substantially between individuals [7,10,11,13].

### 4.1. Vocal Fold Atrophy and Bowing

Age-related reduction in vocal fold bulk is one of the most recognizable features of presbylarynx and is associated with remodeling of both the thyroarytenoid muscle and the lamina propria [7,11]. Endoscopically, it may manifest as thinning of the membranous vocal folds, prominence of the vocal processes, and medial edge bowing, particularly during phonation. Comparison of respiratory and phonatory views can assist in distinguishing the loss of tissue bulk from abnormalities of vocal fold mobility or glottic closure.

Quantitative videostroboscopic approaches indicate that vocal fold bowing exists along a measurable continuum rather than as a simple present-or-absent finding [6,10]. Nevertheless, its assessment remains partly dependent on the viewing angle, image quality, phonatory task, and examiner judgment.

Glottic insufficiency may occur when tissue loss prevents complete medial approximation during phonation. The resulting gap may have an anterior or spindle-shaped configuration [21,22]. Such insufficiency may increase transglottal airflow and reduce phonatory efficiency, potentially contributing to breathiness, reduced vocal intensity, and decreased phonation time [13,29].

Presbylaryngeal atrophy and bowing are commonly bilateral and relatively symmetrical. Marked asymmetry, unilateral bowing, impaired mobility, or a focal mucosal abnormality should prompt evaluation for alternative or coexisting conditions, including vocal fold paresis or paralysis, scarring, benign vocal fold lesions, and neurogenic disease [13,14]. Compensatory supraglottic constriction may partially conceal the underlying insufficiency or introduce an additional hyperfunctional component of the larynx [16,20,24].

### 4.2. Changes in the Lamina Propria

Histological aging of the vocal folds involves the epithelium, the layered architecture of the lamina propria, and the cells that maintain its extracellular matrix (ECM). Reviews by Mizuta, Kuhn, and Hamilton emphasize the relationship between tissue organization and cellular homeostasis, while distinguishing developmental maturation, adult maintenance, injury-related repair, and aging [30,31,32]. Tissue mechanics depend on matrix organization, hydration, turnover, and composition.

Human histological studies demonstrate considerable variation by tissue layer, sex, and individual. Hirano et al. described intermediate-layer thinning and increased deep-layer collagen density predominantly in men, whereas mucosal and cover thickening were more evident in women; superficial edema occurred in both sexes [33]. Histomorphometry has also identified age-associated changes in lamina propria thickness and reduced epithelial cell density [34]. These observations argue against a uniform pattern of mucosal thinning. Reduced epithelial cell density alone does not establish impaired barrier function [32].

Collagen remodeling includes changes in architecture that may occur independently of changes in total content. Microscopy of aged human vocal folds demonstrated dense collagen bundles, reduced interstitial spaces, irregular fibrils, and occasional loss of the normal layered arrangement [35]. A male cadaver cohort showed increased type I and III collagen and decreased elastin, without corresponding increases in types IV and V [36]. However, other histological studies summarized by Mizuta reported unchanged total collagen between adult and geriatric groups or increased elastin staining with age [32]. Elastic-fiber fragmentation, thickening, and disorganization further indicate that fiber abundance cannot be equated with preserved elasticity [32,37].

Hyaluronic acid contributes to hydration and the viscoelastic properties required for mucosal oscillation [38,39]. Reduced staining has been reported in the superficial and deep lamina propria of older men [40]. Conversely, a biochemical study found substantial variability and no significant age-related difference in hyaluronic acid concentration at the sampled human vocal fold sites [41]. Findings therefore depend on the anatomical compartment, population, and measurement method. Proteoglycans, including decorin and fibromodulin, also contribute to matrix organization, although their specific alterations in naturally aged human vocal folds remain incompletely characterized [30,32].

Resident fibroblasts synthesize and remodel ECM components, while vitamin A-storing stellate cells within the maculae flavae have been implicated in matrix renewal and maintenance [30,42,43]. Ultrastructural studies reported less developed rough endoplasmic reticulum and Golgi apparatus in geriatric macula flava cells, consistent with reduced synthetic activity [43,44]. Aged stellate cells also exhibited degenerative changes, fewer peripheral vesicles, and less newly released matrix material [42]. These findings do not directly quantify synthesis throughout the lamina propria: Awd Allah et al. described different organelle changes in fibroblasts of Reinke’s space, emphasizing regional heterogeneity [44]. Moreover, Carneiro et al. found no significant age-related difference in fibroblast concentration in the maculae flavae or vibrating portion in a small cadaver series [45]. Functional cellular alteration therefore should not be assumed to require a demonstrable reduction in cell number.

Human cell-culture studies provide complementary mechanistic evidence. Chen and Thibeault studied fibroblast lines from donors aged 21, 59, and 79 years and reported reduced proliferation and lower expression of several collagen-related genes in cells from older donors [46]. In a separate single-donor study, serial passaging reduced procollagen I, collagen III, and hyaluronan synthase 2 transcripts, with accompanying changes in matrix-degrading enzyme expression [47]. These findings support altered regulation of matrix production and remodeling. However, culture-induced replicative senescence and limited donor numbers restrict extrapolation to natural aging; cellular senescence remains a proposed contributor rather than a mechanism established by these experiments in the aging human vocal fold [30,47].

Collagen accumulation also does not necessarily indicate increased collagen synthesis. Ohno et al. observed dense collagen deposition in aging rats despite reduced procollagen I and III and matrix metalloproteinase 2 and 9 transcripts, alongside reduced hyaluronan synthase expression and hyaluronic acid deposition [48]. Kawai et al. found increased type I collagen protein in cultured aged rat fibroblasts despite unchanged Col1a1 expression and reduced Mmp1 transcription [49]. Together, these findings suggest an altered balance between matrix production and removal, although transcript abundance and static histology do not directly measure turnover rates.

Experimental modulation of aged cells further supports a role for altered matrix regulation. In cultured rat vocal fold fibroblasts, Hirano et al. reported lower hyaluronic acid production with aging; basic fibroblast growth factor increased hyaluronic acid production and reduced type I collagen production under the tested conditions [50]. These observations demonstrate biological responsiveness in vitro without establishing restoration of aged human mucosa.

Oxidative injury and ferroptosis, an iron-dependent form of cell death associated with lipid peroxidation, are additional candidate pathways. In a rat study, local ferrostatin-1 administration attenuated collagen accumulation and preserved hyaluronic acid distribution in older animals [51]. The short follow-up and absence of phonatory outcome measurements limit interpretation. These findings support further mechanistic investigation but do not establish ferroptosis as a cause of human presbylarynx or ferrostatin-1 as a clinical treatment.

**Figure 2:**
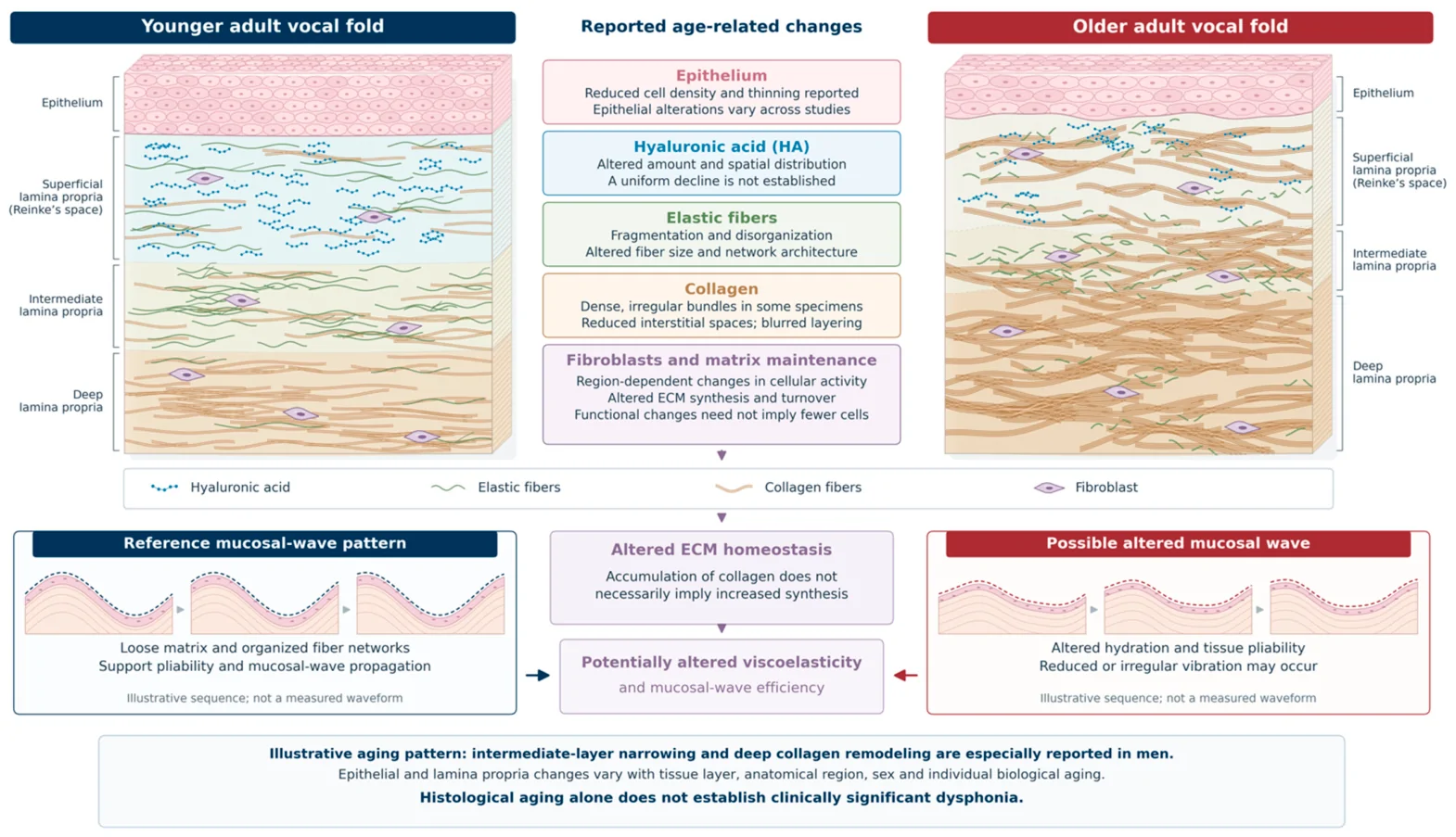
Age-related changes in the vocal fold lamina propria and their potential biomechanical consequences. Original schematic created by the authors from human histological and experimental evidence [33,34,35,37,38,41,44,45,48]. Reported epithelial, matrix, and fibroblast changes are shown alongside possible effects on mucosal vibration. Patterns vary by layer, sex, and individual; uniform hyaluronic acid loss is not implied. Drawings are not to scale; waveforms are illustrative. ECM, extracellular matrix; HA, hyaluronic acid.

ECM remodeling may affect mucosal-wave propagation, but vibratory abnormalities are nonspecific and also occur in scarring and other mucosal disorders [11,13,30,37,38]. Videostroboscopy cannot identify a particular ECM or fibroblast abnormality, and histological aging alone does not establish clinically meaningful dysphonia. Figure 2 summarizes the potential biomechanical consequences.

### 4.3. Thyroarytenoid Muscle Aging

The thyroarytenoid muscle is an important component of the vocal fold body and contributes to vocal fold bulk, medialization, tension regulation, and fine control of phonation. Human muscle-fiber and connective-tissue findings are summarized in Table 2. Mitochondrial accumulations and ragged-red fibers have also been observed in aged human thyrovocalis specimens, suggesting a myopathic component to muscle aging [52].

The pattern of fiber-type changes in the aging thyroarytenoid muscle should not be assumed to mirror conventional limb muscle sarcopenia [53]. A quantitative human study identified an age-related reduction in the length density of Type I slow-twitch fibers, increased slow–fast myosin coexpression, and a greater frequency of very small Type II fibers [54]. In contrast, Kersing and Jennekens described Type I predominance and smaller Type II fibers in older thyrovocalis specimens, with little fiber-type grouping [52]. Thus, fiber-type grouping is not a consistent finding and remains indirect evidence of denervation–reinnervation rather than proof of its extent or cause. Variations among studies may reflect differences in age distribution, sex, sampling location, histological methods, and the complex regional organization of the thyroarytenoid muscle [52,53,54,55].

In a cross-sectional MRI study of 44 adults, thyroarytenoid dimensions and estimated volume were negatively associated with age in models including age and sex [56]. Volume was approximated from linear dimensions rather than full volumetric segmentation; these findings do not establish longitudinal rates of muscle loss. Sex-related findings and the separate electromyographic cohort are discussed in Section 4.5.

Animal experiments provide more direct evidence of altered contractile performance. In Fischer 344 × Brown Norway rats, McMullen and Andrade reported lower thyroarytenoid twitch and tetanic forces at 30 months, slower shortening, and reduced fatigue resistance with advancing age, together with mitochondrial and glycogen alterations [57]. These findings cannot be directly extrapolated to human presbyphonia.

Animal findings also caution against equating aging with uniform myofiber atrophy. Bhatt et al. found greater mean thyroarytenoid myofiber cross-sectional area in 21-month-old than in 4-month-old rats, despite prolonged evoked compound muscle action potential latency [58]. Differences in bowing and recurrent laryngeal nerve myelination did not reach statistical significance. Myofiber size, total muscle volume, and contractile performance are distinct outcomes; larger fibers alone do not establish preserved function or exclude remodeling elsewhere in the vocal fold.

In aging human thyroarytenoid muscle, an increased relative length of fibers expressing developmental myosin suggests retained regenerative activity, although this may not compensate fully for structural remodeling [59]. Injury recovery is a distinct process: after bupivacaine-induced injury, older rats showed less recovery of myofiber cross-sectional area at 10 days, without a significant age difference in early satellite-cell mitotic activity [60]. This finding does not establish generalized satellite-cell failure or impaired spontaneous regeneration in older humans.

Residual plasticity was also demonstrated in aged sheep, in which 29 days of unilateral recurrent laryngeal nerve stimulation increased thyroarytenoid fiber diameter without significantly changing fiber-type distribution. Human phonatory benefit remains unestablished [61].

Reduced muscle reserve may increase susceptibility to vocal fatigue and reliance on compensatory phonatory strategies, particularly during prolonged or high-demand voice use. Structural findings should therefore be interpreted alongside functional assessment rather than used alone to infer clinically significant dysphonia [53,57]. Table 2 summarizes the principal morphological alterations reported in the aging thyroarytenoid muscle and their potential—not deterministic—functional implications.

**Table 2:** Principal age-related alterations of the thyroarytenoid muscle and neuromuscular apparatus and their potential functional implications.

Age-Related Process	Morphological and Neuromuscular Findings	Potential Functional Implications
Muscle fiber atrophy and loss	• Reported reductions in fiber diameter and cross-sectional area; findings vary by study and model • Age-related loss of muscle fibers • Decreased thyroarytenoid muscle bulk	• Reduced vocal fold bulk • Contribution to vocal fold atrophy, bowing, and glottic insufficiency • Reduced contractile strength and phonatory reserve
Muscle fiber-type remodeling	• Age-related changes in Type I and Type II fiber distribution • Variably reported fiber-type grouping and myosin coexpression, compatible with motor-unit remodeling • Direction and magnitude differ among studies	• Potential alteration of contraction speed and fatigue resistance • Reduced precision of rapid tension adjustment • Clinical significance remains incompletely defined
Muscle fiber injury and regeneration	• Age-associated increase in the relative length of fibers expressing developmental myosin • Possible compensatory response to increased fiber injury or death • May not fully offset age-related structural remodeling	• Partial maintenance of muscle mass and function • Reduced capacity to compensate for continuing muscle injury • Potential reduction in functional reserve during demanding phonation
Denervation, reinnervation, and neuromuscular-junction remodeling	• Human histological patterns compatible with partial denervation–reinnervation • Motor-unit remodeling and variably reported fiber-type grouping • Denervation-like neuromuscular-junction alterations in aged animal models	• Reduced precision and stability of vocal fold movement • Increased variability of phonatory output • Greater vulnerability to vocal fatigue and functional decompensation

Functional effects are potential, not inevitable. Evidence includes human histological and animal studies; detailed age-related NMJ alterations are supported mainly by animal models. Fiber-size findings vary by study and model, and Type I/II fiber redistribution remains inconsistent across human studies [52,54,55,57,58,59,60,62,63,64,65,66]. TA, thyroarytenoid; NMJ, neuromuscular junction.

### 4.4. Neuromuscular and Neural Aging

Human nerve studies also suggest selective rather than uniform age effects. Mortelliti et al. found preferential loss of small, myelinated fibers in the superior laryngeal nerve [64]. Tiago et al. reported fewer myelinated fibers in the recurrent laryngeal nerve of older cadavers, without a significant difference in total superior laryngeal nerve counts [66]. These findings support neural involvement but should not be generalized to equivalent degeneration across all laryngeal nerves and fiber populations. Such remodeling may preserve gross vocal fold mobility while reducing the precision, coordination, and adaptive reserve of laryngeal muscle activation.

Human electrophysiological evidence is consistent with motor unit remodeling. In 26 healthy adults aged 21–72 years, Takeda et al. found longer thyroarytenoid motor unit action potential durations in participants older than 60 years, with substantial variability among older individuals [65]. This pattern is compatible with denervation–reinnervation and enlargement of surviving motor units. However, these normative, cross-sectional observations do not establish pathological denervation in every older adult, identify a diagnostic age threshold for presbyphonia, or directly demonstrate impaired neuromuscular transmission.

Aged rat thyroarytenoid neuromuscular junctions show smaller axon-terminal areas, uncovered postsynaptic receptor regions, and greater endplate variability [62]. In rat thyroarytenoid and posterior cricoarytenoid muscles, age-related reductions in endplate size and density accompanied increased sensitivity to tubocurarine, indicating reduced transmission reserve under experimental blockade without localizing a single presynaptic or postsynaptic defect [63]. These findings provide mechanistic evidence rather than direct confirmation of equivalent changes in older humans.

Central changes have also been reported. Basken et al. found fewer thyroarytenoid-projecting neurons in the nucleus ambiguus of aged rats alongside call-dependent reductions in ultrasonic vocalization bandwidth and intensity; causality was not established [67]. In age-group comparisons, Fogarty found dendritic changes and altered swallowing timing before detectable reductions in swallowing pressure and motor neuron counts. Because swallowing was assessed under anesthesia, these findings do not establish the sequence underlying human presbyphonia [68].

The independent contributions of neural and neuromuscular aging to human voice impairment remain incompletely defined [63,65,67,68]. However, vocal instability and tremor are not specific manifestations of presbylarynx and should prompt consideration of neurological, neurodegenerative, and other laryngeal disorders when clinically prominent [13,14].

### 4.5. Sex-Related Differences

Sex-related differences should be considered at the tissue and neuromuscular levels as well as in relation to endocrine status. Human histological studies have described more pronounced membranous vocal fold shortening, intermediate-layer thinning and elastic-fiber loss, and deep-layer collagenous remodeling in older men, whereas mucosal and cover thickening were more prominent in older women. Superficial lamina propria edema occurred in both sexes, with substantial individual variability [33]. Hammond et al. also found less collagen in adult and geriatric female vocal folds than in male specimens, without a significant overall difference between adult and geriatric groups [69]. Thus, sex-associated differences in tissue composition should be distinguished from evidence of progressive age-related change.

Muscle size and electrophysiological findings provide complementary, but non-equivalent, information. In an MRI study of 44 adults, Malkidou et al. reported larger thyroarytenoid and cricothyroid dimensions and estimated volumes in men, with negative age associations for thyroarytenoid dimensions and for cricothyroid thickness and volume. In a separate cohort of 29 volunteers aged 21–64 years, surface recordings over the cricothyroid region showed higher phonatory activation amplitudes in women, while increasing age was associated with lower activation amplitudes. Surface recordings, the limited age range, and the use of independent MRI and electromyographic cohorts constrain interpretation and do not establish a relationship between muscle size and activation in the same individual [56]. Moreover, needle electromyography of the thyroarytenoid muscle in 26 healthy adults identified age-related prolongation of motor unit potentials without a significant difference in duration between men and women [65]. These findings do not establish a consistent sex-specific pattern of laryngeal denervation or neuromuscular transmission failure.

Sex should therefore inform interpretation of laryngeal morphology and functional measurements alongside age, body size, endocrine status, and communication demands. Differences in absolute tissue or muscle measurements do not, by themselves, demonstrate different rates of aging or predict individual voice disability. Longitudinal studies with sex-stratified analyses and explicit testing of sex-by-age interactions are needed to clarify whether structural and neuromuscular trajectories differ and whether such differences influence treatment response.

## 5. Functional Consequences of Presbylarynx

Presbylaryngeal morphology may be associated with a broad spectrum of functional voice changes. However, functional impairment is not an inevitable consequence of structural aging of the larynx. Voice outcomes reflect the interaction between vocal fold morphology, neuromuscular control, respiratory support, compensatory capacity, systemic health, and individual communication demands [13,16,20,29].

### 5.1. Voice Quality Changes

Common perceptual features include breathiness, reduced loudness, weakness, roughness, and instability [13,29,70,71]. Glottic insufficiency may increase airflow and reduce phonatory efficiency, but these features are nonspecific and also occur in structural, inflammatory, neurogenic, and functional disorders [13,14,29]. Vocal fatigue and difficulty sustaining or projecting the voice may become apparent during prolonged speech or communication in noise [13,29].

**Figure 3:**
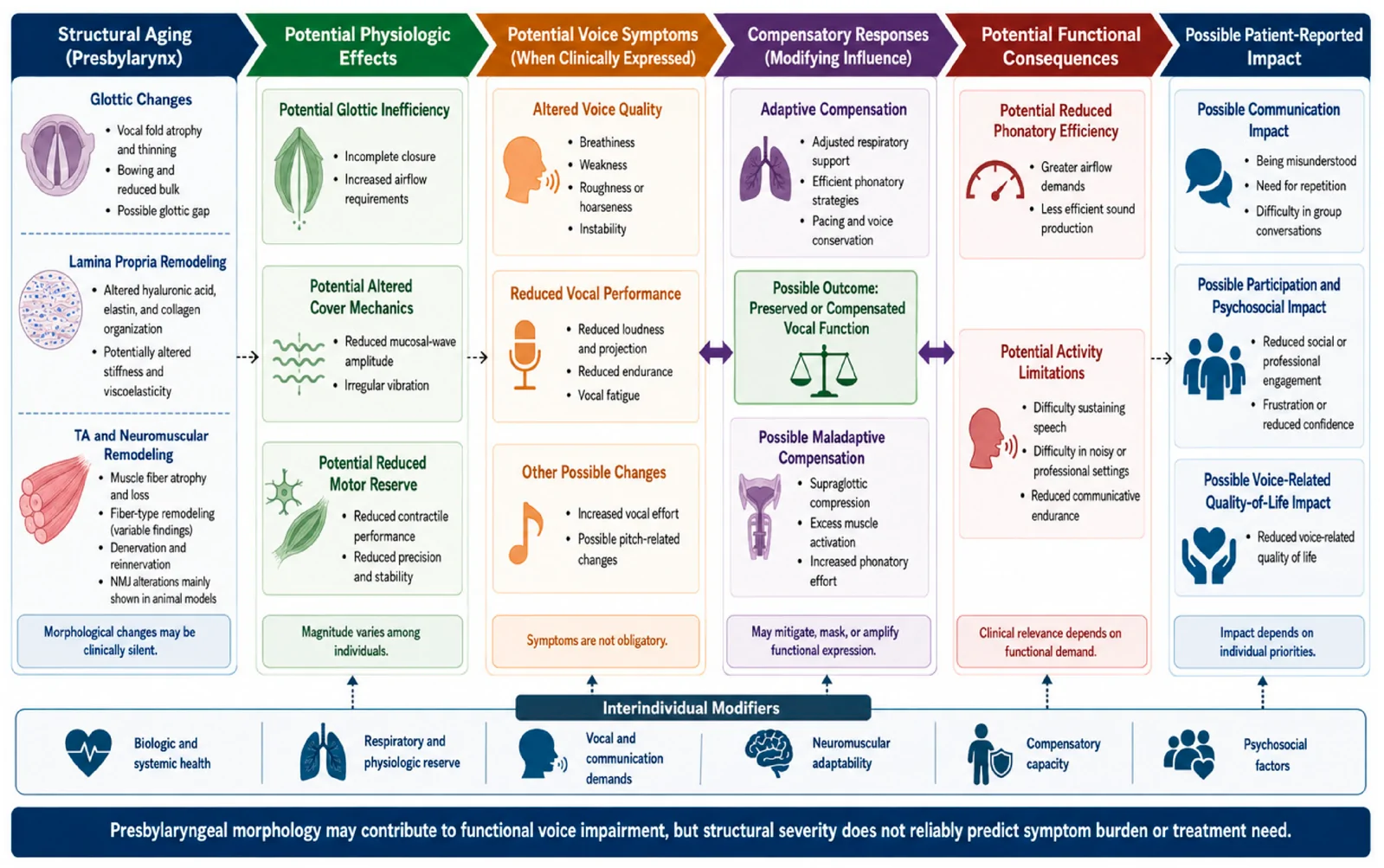
Potential relationships between presbylaryngeal morphology and functional voice outcomes. Original schematic created by the authors, synthesizing structural, neuromuscular, respiratory, and clinical observations [11,16,19,38,54,62]. Dashed arrows indicate potential associations; bidirectional arrows represent interactions with compensatory responses; upward arrows show interindividual modifiers. Morphology alone does not determine voice impairment or treatment need. TA, thyroarytenoid; NMJ, neuromuscular junction.

Loudness studies have reported lower sound pressure levels and altered laryngeal muscle activation in older adults despite comparable subglottal pressure, indicating that reduced loudness cannot be attributed solely to respiratory limitation [72]. The resulting burden depends on communication demands, compensatory capacity, and social context; vocal aging may also influence listener perceptions [73]. Consequently, marked morphological changes may produce little perceived impairment, whereas subtle findings may accompany substantial limitations [16,20,74]. Figure 3 summarizes these potential relationships.

### 5.2. Acoustic and Aerodynamic Findings

Older speakers may show changes in perturbation, noise-related, spectral, and cepstral measures, but findings vary by sex, task, recording conditions, software, and population and may not correspond to perceptual or patient-reported impairment [17,70,71]. Speaking fundamental frequency generally rises in older men, whereas women may show different trajectories, particularly after menopause; endocrine status, tissue properties, and vocal behavior also influence these patterns [71,75,76].

Functionally relevant glottic insufficiency may produce increased airflow, shortened maximum phonation time, and reduced phonatory efficiency, with additional effects from pulmonary mechanics and respiratory reserve [13,19,29,77]. Because values overlap between symptomatic and asymptomatic older adults and other voice disorders, acoustic and aerodynamic findings require interpretation alongside history, laryngeal imaging, perceptual assessment, and patient-reported outcomes [13,17,29,71].

### 5.3. Compensatory Mechanisms

Compensatory changes in vocal fold adduction, supraglottic recruitment, respiratory drive, and phonatory effort may preserve communication despite structural aging [13,29]. Supraglottic constriction may support voice production but can also increase effort, fatigue, or discomfort. It is not necessarily secondary compensation and may reflect a coexisting functional disorder, another laryngeal abnormality, or task-dependent behavior [78,79].

Hyperfunction may obscure glottic insufficiency, while prominent constriction can distract from structural or neurogenic contributors. Assessment across phonatory tasks and intensities helps distinguish adaptive strategies from maladaptive behavior that may warrant behavioral intervention [78,79,80].

## 6. Interaction Between Presbylarynx and Systemic Aging

Age-related changes in physiological reserve, respiratory capacity, endocrine function, neuromuscular control, cognitive status, and general health may modify the functional relevance of presbylaryngeal morphology. These factors may also contribute to dysphonia, independent of laryngeal aging. Therefore, they should be interpreted as potential modifiers or coexisting causes rather than evidence that presbylaryngeal morphology alone is responsible for a patient’s voice impairment.

### 6.1. Frailty and Physiological Aging

Frailty is a state of reduced physiological reserve and increased vulnerability, commonly characterized by weight loss, exhaustion, weakness, slow walking speed, and low activity [81]. Presbylaryngeal findings have been associated with comorbidity burden and functional dependence, but direct evidence linking morphology to frailty remains limited [12]. In another study, voice-related handicap and cognitive status differed between frailty groups, whereas acoustic measures did not consistently differ; exhaustion and weight loss were particularly related to self-reported handicap [18].

Frailty may help contextualize respiratory reserve, endurance, compensatory capacity, and treatment feasibility. However, neither presbylaryngeal morphology nor frailty assessment alone is a validated diagnostic marker of presbyphonia or predictor of individual voice outcomes [12,18].

### 6.2. Respiratory System Aging

Age-related changes in pulmonary elasticity, chest-wall compliance, respiratory muscle performance, and breathing regulation may alter speech-breathing and respiratory–laryngeal coordination [82]. When combined with glottic air leakage, reduced reserve may shorten phrases and increase effort or fatigue; performance also depends on laryngeal valving and behavioral adaptation [19,72]. Respiratory aging is a potential contributor, but its relative importance remains incompletely characterized, and it is not a universal explanation for presbyphonia [77].

Assessment should consider respiratory symptoms, exercise tolerance, pulmonary disease, inhaler use, speech-breathing behavior, and sustained phonation or loudness. Targeted pulmonary evaluation may be appropriate when the history or examination suggests respiratory limitation [77].

### 6.3. Endocrine and Hormonal Influences

Endocrine factors may influence vocal tissue hydration, matrix composition, and muscle function [83,84,85,86]. Menopause has been associated with changes in fundamental frequency, stability, flexibility, and effort, whereas direct evidence linking androgen decline to presbylaryngeal atrophy or severity remains limited [75,83,86,87]. Human vocal fold hormone-receptor findings vary by tissue and method and support biological plausibility without establishing a causal role in presbylaryngeal morphology or symptoms [88].

Thyroid dysfunction, growth hormone deficiency, and metabolic disease may alter voice independently of laryngeal aging [84,85,89,90]. Improvement following thyroid replacement in some patients highlights a potentially treatable contributor but does not demonstrate reversal of presbylarynx [84,91]. Endocrine assessment should therefore be guided by the clinical history, associated symptoms, and examination findings.

### 6.4. Neurological and Cognitive Factors

Aging may alter motor timing, sensorimotor integration, and auditory–motor regulation, while peripheral neural and motor-unit remodeling may reduce laryngeal adaptability [64,65,66,92,93]. However, much cognitive-aging research concerns articulation, fluency, rhythm, and connected speech rather than laryngeal voice production; these associations do not establish cognitive decline as a cause of presbylarynx [92,93].

Hypophonia, tremor, asymmetrical movement, or impaired coordination may indicate coexisting neurological disease and require differential assessment (Section 7.4) [94,95,96,97]. Neurological and cognitive evaluation should be targeted to the history, communication profile, examination, and treatment demands. Cognitive impairment may affect symptom reporting, test performance, and learning; therapy goals and home practice should reflect the patient’s capacity and available support [18,92,93,98].

### 6.5. Systemic Diseases and Medications

Dysphonia in older adults is often multifactorial, reflecting systemic health, coexisting laryngeal disease, functional status, and medication exposure [12,89,99,100,101]. Cardiopulmonary disease may reduce vocal endurance through limited respiratory reserve, while diabetes has been associated with nonspecific subjective and objective voice abnormalities [19,77,85,102]. Endocrine contributors are discussed in Section 6.3.

A case–control study in men with Reinke’s edema did not confirm an association with hypothyroidism; its findings do not directly address presbylaryngeal aging [103]. Medications may affect voice through xerostomia, reduced lubrication, irritation, or altered neuromuscular function [104,105]. Anticholinergic and psychotropic agents are relevant to cumulative xerogenic burden [106,107]. Inhaled corticosteroids may cause dysphonia through local deposition, mucosal irritation, candidiasis, or laryngeal myopathy [108,109,110]. New symptoms require review of timing, technique, dose, and laryngeal findings; medication changes should be coordinated with the prescribing clinician.

**Figure 4:**
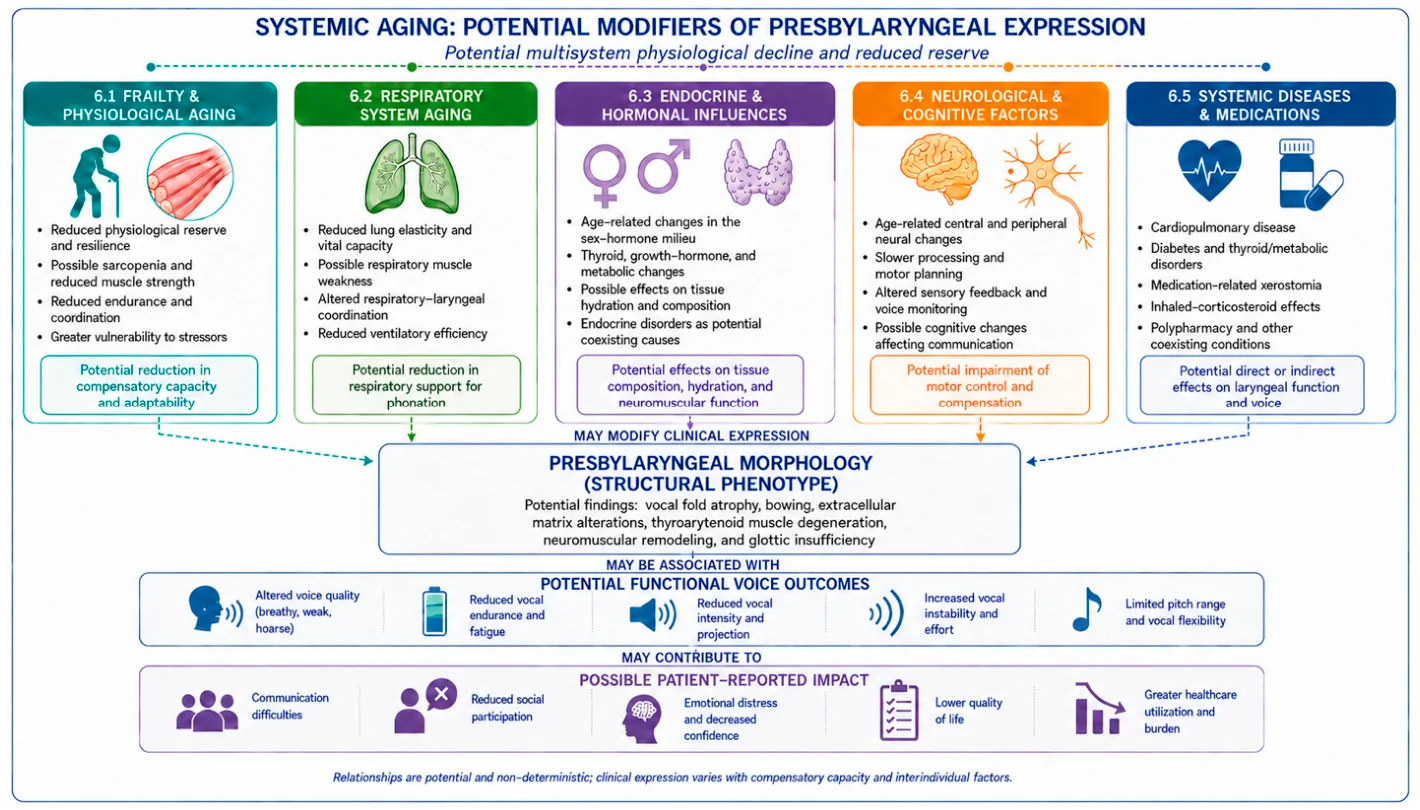
Potential systemic modifiers of presbylaryngeal clinical expression. Original schematic created by the authors, integrating evidence on comorbidity, endocrine status, respiratory function, frailty, neurological and cognitive function, and medication effects [12,18,19,84,92,93,104,108]. These factors may influence voice function and compensatory capacity. Dashed arrows represent proposed, non-deterministic relationships and do not establish direct causality.

A targeted comorbidity and medication review helps identify treatable contributors and factors affecting treatment feasibility and response [12,18,19,84,93,102,104,108]. Figure 4 summarizes these potential systemic modifiers.

## 7. Differential Diagnosis of Dysphonia in Older Adults

The differential diagnosis of dysphonia in older adults is challenging because age-related laryngeal morphology, systemic disease, neurological dysfunction, focal laryngeal pathology, and compensatory phonatory behaviors frequently coexist. Similar symptoms and endoscopic appearances may occur with benign or malignant vocal fold lesions, inflammatory disease, vocal fold paresis or paralysis, neurogenic dysphonia, and functional voice disorders [80,89,99,111,112].

The presbylarynx should be understood as a descriptive morphological phenotype rather than a diagnosis of exclusion. The diagnostic step requiring exclusion is the attribution of clinically meaningful dysphonia to presbylaryngeal changes. In other words, age-related morphology may support a diagnosis of presbyphonia only when the patient’s symptoms and functional findings are concordant, and alternative or coexisting causes have been adequately evaluated. This distinction is important because clinical studies of older adults with dysphonia have frequently identified pathological or multifactorial explanations rather than physiological aging alone [89,99,112,113].

### 7.1. General Diagnostic Principles

Evaluation combines symptom history and red-flag assessment, laryngeal imaging, and multidimensional voice measures; the assessment domains are detailed in Section 8 [17,80,111]. Presbyphonia should be considered when symptoms and functional findings are compatible with age-related voice impairment after alternative and coexisting causes have been evaluated. No single morphological or voice measure establishes this attribution [17,23,111,112].

**Figure 5:**
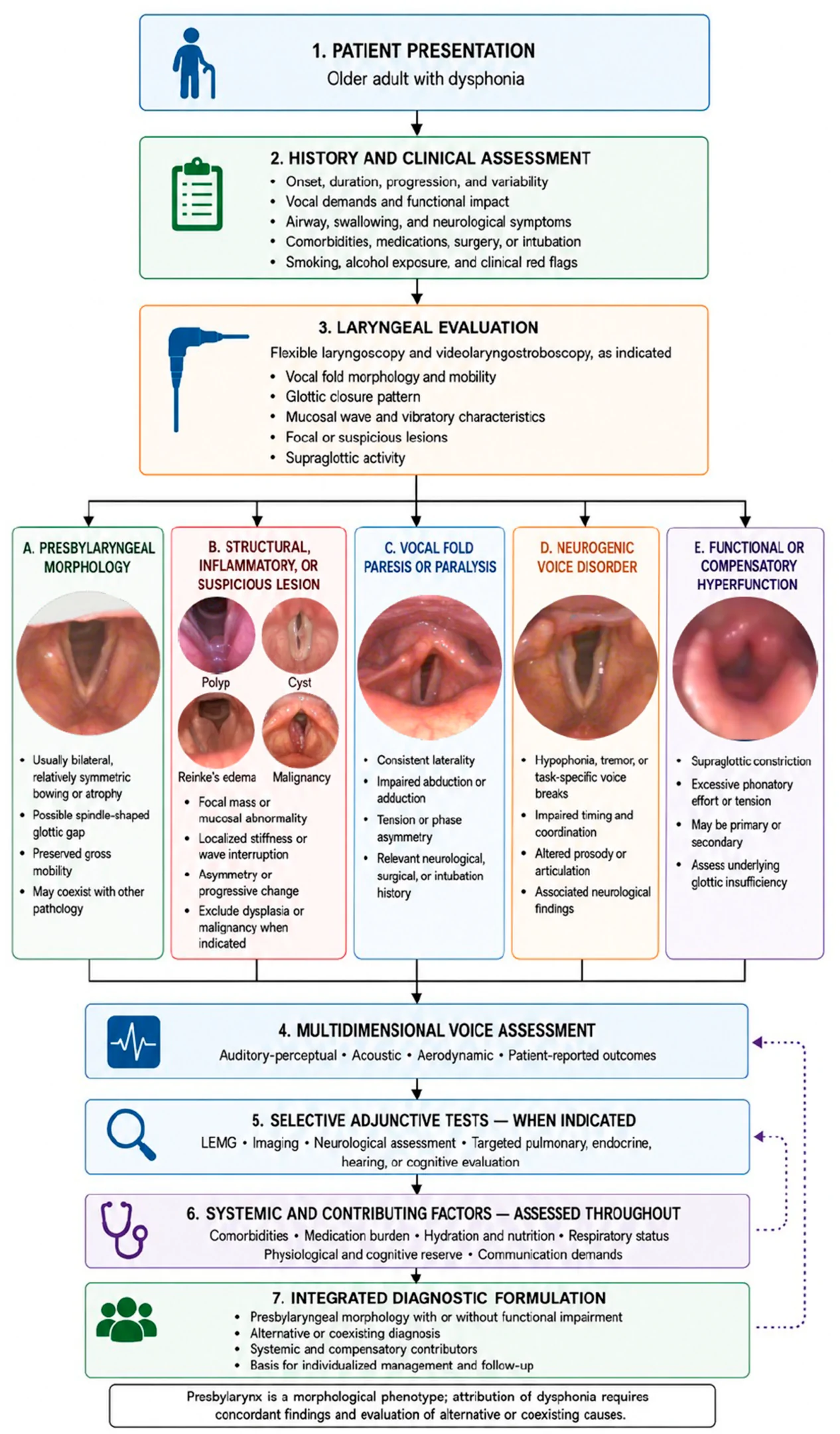
Proposed multidimensional diagnostic framework for dysphonia in older adults. Original schematic created by the authors, informed by voice-assessment recommendations and clinical evidence [17,111,112,114]. History, laryngeal examination, multidimensional voice assessment, systemic factors, and selective adjunctive testing inform the differential diagnosis. Illustrative endoscopic appearances do not establish diagnoses independently. The framework requires clinical judgment and is not a validated diagnostic algorithm. LEMG, laryngeal electromyography.

Adjunctive tests should address a specific diagnostic question: laryngeal electromyography (LEMG) may help evaluate paresis or distinguish neurogenic dysfunction from mechanical fixation, while imaging may be indicated for suspicious lesions or unexplained immobility. Neurological, pulmonary, endocrine, hearing, or cognitive assessment should be selected according to clinical findings [14,111,114,115]. Figure 5 summarizes the proposed diagnostic framework.

### 7.2. Presbylarynx Versus Structural and Inflammatory Laryngeal Disorders

Benign vocal fold lesions, inflammatory conditions, epithelial abnormalities, scarring, and neoplastic diseases are important causes of dysphonia in older adults. Accordingly, the identification of a focal lesion does not exclude background presbylaryngeal morphology, and the presence of presbylarynx does not establish that a coexisting lesion is incidental [2,99,111,112,113].

Presbylaryngeal changes are generally characterized by diffuse loss of vocal fold bulk, frequent bilateral bowing, prominence of the vocal processes, and variable glottic insufficiency. Benign structural lesions more often produce focal or regionally asymmetric alterations in mass, contour, or stiffness. Polyps, cysts, nodules, and Reinke’s edema may share perceptual features with presbyphonia. Perceptual voice quality alone is therefore insufficient for differentiation [80,112,116,117].

Videolaryngostroboscopy (VLS) is particularly useful for evaluating whether a visible abnormality produces a localized disturbance in vibration. Findings that favor a focal lesion include a discrete mass, segmental reduction or interruption of the mucosal wave, localized stiffness, asymmetric vibration, and a closure pattern anatomically related to the lesion. However, vibratory amplitude and mucosal-wave behavior may vary, and bilateral symmetry should not be treated as an absolute diagnostic criterion [10,80,118,119,120].

Mucosal edema, erythema, surface irregularity, secretions, and discomfort may coexist with age-related atrophy and alter glottic closure and vibratory behavior. Interpret nonspecific inflammatory findings in relation to symptoms, exposure history, medication use, and other examination findings, rather than automatically attributing them to reflux or aging. Persistent or progressive unilateral abnormalities, epithelial irregularity, leukoplakia, ulceration, impaired mobility, or unexplained focal stiffness require appropriate investigation to exclude dysplasia or malignancy, particularly in patients with relevant exposure histories [111,112].

The clinical significance of each abnormality should be determined by concordance among lesion location, vibratory disturbance, perceptual findings, and symptom patterns. A small focal lesion may not account for severe breathiness when substantial bilateral atrophy and glottic insufficiency are present. Conversely, diffuse presbylaryngeal morphology should not be used to dismiss focal lesions that produce localized stiffness or progressive symptoms. This distinction is also relevant when counseling patients about treatment: lesion-directed surgery may correct focal pathology but may not eliminate residual dysphonia arising from persistent glottic insufficiency, reduced physiological reserve, or compensatory hyperfunction [80,112,117].

### 7.3. Presbylarynx Versus Vocal Fold Paresis or Paralysis

Presbylarynx and vocal fold paresis or paralysis may present with breathy or weak voice quality, reduced vocal intensity, vocal fatigue, shortened maximum phonation time, and incomplete glottic closure. Partial recurrent or superior laryngeal nerve dysfunction may be especially difficult to distinguish from presbylaryngeal change because mild paresis can produce bowing, reduced vocal fold bulk, altered tension, and compensatory supraglottic activity without complete immobility [14,80,114,116].

Age-related thyroarytenoid muscle atrophy, changes in muscle-fiber composition, motor-unit remodeling, and peripheral neural aging may create an appearance suggestive of mild weakness even in the absence of a discrete neural lesion [54,55,64,65,66]. Conversely, unilateral paresis may be mistaken for asymmetric aging. Consequently, the diagnosis should be based on a reproducible pattern of neurological dysfunction rather than bowing or glottic insufficiency alone.

Findings that increase the suspicion of paresis or paralysis include consistent laterality across phonatory and respiratory tasks, reduced or absent abduction or adduction, asymmetric vocal fold elongation or tension, persistent phase asymmetry, arytenoid positional asymmetry, and unilateral compensatory supraglottic activity. A history of abrupt onset, neck or thoracic surgery, intubation, malignancy, viral illness, or associated swallowing and airway symptoms may provide additional diagnostic information. In contrast, presbylaryngeal atrophy is more often bilateral and relatively symmetric, although considerable interindividual variation exists and symmetry alone cannot exclude neurogenic dysfunction [14,80,114,116,117].

LEMG can provide complementary evidence when clinical and endoscopic findings remain inconclusive or when the results are expected to alter management. It may help identify denervation or reinnervation, differentiate neurogenic dysfunction from mechanical fixation, and provide prognostic information in confirmed vocal fold paralysis [114,115]. However, LEMG should not be routinely used to confirm presbylarynx. In a retrospective cohort of voice patients investigated for suspected paresis, LEMG recruitment did not differ significantly between patients with presbylarynx and comparison groups without presbylarynx. These findings do not establish normal neuromuscular function in presbylarynx or support using morphology alone as evidence of denervation [121].

Interpretation of LEMG in older adults should also account for age-related neural and motor-unit remodeling, technical variability, muscle selection, timing after suspected nerve injury, and the possibility of partial or recovering lesions [64,66,114,115,121].

### 7.4. Presbylarynx Versus Neurogenic Dysphonia

Neurogenic voice disorders become increasingly relevant with advancing age and may coexist with presbylaryngeal morphology in older adults [122]. Parkinson’s disease, essential or dystonic tremor, laryngeal dystonia, stroke-related dysfunction, motor-neuron disease, and other central or peripheral neurological disorders may produce voice symptoms that overlap with presbyphonia [94,95,96,97,123,124]. Endoscopic identification of vocal fold atrophy does not exclude a neurogenic disorder, particularly when the clinical presentation includes abnormalities extending beyond glottic closure.

Parkinsonian voice and speech commonly include reduced loudness, monopitch, monoloudness, diminished prosodic variation, imprecise articulation, altered speech rate, and impaired respiratory–phonatory coordination. Vocal tremor, breathiness, roughness, and glottic insufficiency may also occur [96,123,124,125].

Patterns of initiation, timing, coordination, task specificity, and rhythmic stability are often more informative than the presence of glottic gaps. Presbylaryngeal insufficiency primarily reflects reduced bulk and altered tissue or muscle properties, whereas neurogenic disorders may result in impaired scaling, delayed initiation, rhythmic oscillation, task-dependent voice breaks, or inconsistent motor control. Associated dysarthria, articulation changes, dysphagia, facial or limb motor findings, gait disturbance, tremor, cognitive change, and autonomic symptoms further increase suspicion of neurological disease [94,95,96,125].

Acoustic analysis may identify reduced intensity, increased perturbation, altered fundamental frequency variability, or abnormalities in rate and pause distribution. Patients with Parkinson’s disease without subjective voice complaints may already demonstrate measurable acoustic abnormalities, illustrating that objective changes may precede symptom recognition but cannot independently establish a neurological diagnosis [124]. Similarly, correlations between acoustic speech characteristics and general motor impairment vary across tasks and individuals [123].

Laryngeal tremors and dystonia require assessment across different vocal tasks. Rhythmic oscillation, task-specific phonatory breaks, and differential performance during voiced and voiceless sentence tasks may suggest a neurogenic mechanism. However, muscle tension dysphonia can mimic laryngeal dystonia, and no isolated perceptual or endoscopic feature provides perfect diagnostic separation [97]. When central motor dysfunction is suspected, consider neurological evaluation and multidisciplinary assessment rather than attributing the presentation to presbylaryngeal aging alone.

### 7.5. Functional Voice Disorders and Compensatory Hyperfunction

Functional voice disorders, particularly muscle tension dysphonia, may occur as primary disorders or develop secondarily as compensatory responses to glottic insufficiency, vocal fold atrophy, paresis, focal lesions, or other laryngeal conditions [78,79,80,99]. In older adults with presbylarynx, increased supraglottic activity may represent an attempt to maintain loudness or stability despite reduced vocal fold bulk. This should not be automatically interpreted as either primary muscle tension dysphonia or an intrinsic component of the presbylarynx.

The principal diagnostic question is whether hyperfunction represents the primary mechanism of dysphonia, a secondary response to an underlying glottic deficit, or a combination. Misclassifying secondary compensation as primary muscle tension dysphonia may lead to behavioral treatment that does not adequately address the underlying glottic insufficiency. Conversely, interpreting all hyperfunction as an unavoidable consequence of aging may overlook modifiable behavioral components [78,79].

Evaluation should include observation of sustained vowels, connected speech, changes in pitch and loudness, and other tasks designed to reveal consistency or task specificity. Palpatory evidence of excessive extrinsic tension, changes with unloading or facilitative maneuvers, and responses to carefully selected behavioral probes may provide additional information. However, temporary improvement during therapeutic maneuvers does not exclude underlying structural or neurogenic disorders [79,97].

Stroboscopy remains important for identifying glottic insufficiency, focal stiffness, or asymmetry, which may be partly obscured by supraglottic constriction [78,79,80].

The final diagnosis should describe both the underlying substrate and the functional response whenever both are clinically relevant, for example, presbylaryngeal atrophy with glottic insufficiency and secondary muscle tension dysphonia, rather than forcing the presentation into a single mutually exclusive category.

## 8. Clinical Assessment: From Morphology to Decision-Making

Building on the differential diagnostic principles in Section 7, clinical assessment integrates laryngeal morphology, functional voice findings, patient-reported impact, and systemic health to determine whether age-related changes contribute meaningfully to dysphonia and whether intervention is warranted [16,17,20,80,111]. The following domains support this assessment.

### 8.1. Medical History

A detailed medical history provides the clinical context required to interpret the presbylaryngeal morphology. The assessment should characterize the onset, duration, trajectory, and variability of voice change, together with vocal fatigue, phonatory effort, reduced projection, instability, and the effect of prolonged voice use. Gradual loss of vocal intensity and endurance may be compatible with age-related voice impairment, whereas sudden onset, rapid progression, marked fluctuation, or prominent asymmetry should prompt consideration of alternative diagnoses [80,98,111,116,126].

Dysphagia, aspiration, dyspnea, persistent cough, odynophonia, hemoptysis, unexplained weight loss, referred otalgia, and neck masses should not be attributed to aging without further evaluation. A history of substantial tobacco or alcohol exposure, previous head and neck malignancy, neck irradiation, airway surgery, prolonged intubation, or recent cervical or thoracic surgery may also alter the differential diagnoses. Progressive or persistent dysphonia accompanied by these features requires timely laryngeal assessment and, when indicated, additional investigation [111].

Targeted neurological questioning should address tremor, bradykinesia, gait disturbance, reduced facial expression, dysarthria, cognitive change, and other symptoms suggesting central or peripheral neurological diseases. Respiratory symptoms, exercise tolerance, and the ability to sustain phonation should also be documented because reduced pulmonary reserve may contribute to diminished vocal intensity and endurance independently of laryngeal morphology [19,82,94,95].

The functional relevance of voice change partly depends on the patient’s communication demands. Therefore, explore occupational voice use, caregiving responsibilities, social participation, telephone communication, speaking in noise, and hearing impairment [80,113]. Assessment should also establish the patient’s priorities and expectations, including whether the primary concern is intelligibility, projection, endurance, effort, or social and emotional consequences of voice change.

Comorbidities and medication exposure require systematic review (Section 6) [84,102,104,106,108]. Hydration and nutritional status, frailty, physical activity, smoking, and alcohol exposure should also be considered. Medical history generates hypotheses that guide subsequent assessment, but it does not, by itself, establish whether presbylaryngeal morphology is responsible for the patient’s symptoms.

### 8.2. Laryngeal Imaging and the Role of Videolaryngostroboscopy

Laryngeal visualization is a central component in the evaluation of persistent dysphonia in older adults. Flexible or rigid laryngoscopy allows the assessment of vocal fold morphology and mobility, glottic closure, supraglottic activity, mucosal abnormalities, secretion management, and focal or suspicious lesions. Findings associated with presbylarynx may include bilateral reduction in vocal fold bulk, membranous bowing, prominence of the vocal processes, and incomplete glottic closure, sometimes producing a spindle-shaped gap [13,22,80,116,120,126]. These findings may occur in different combinations and with varying degrees of asymmetry.

No individual endoscopic sign is specific to the presbylarynx. Age-related atrophy or tissue remodeling may occur without an obvious glottic gap. A relatively symmetric and diffuse pattern may support an age-related morphological interpretation but does not exclude coexisting pathology or demonstrate that the observed changes are the principal cause of dysphonia [6,13,20,22,80,120].

VLS, when available and clinically indicated, adds information about glottic closure, vibratory amplitude, mucosal-wave propagation, phase symmetry, periodicity, and non-vibrating segments. It can help identify subtle mucosal abnormalities and distinguish diffuse atrophic changes from focal lesions or unilateral dysfunction. However, VLS remains a sampling technique based on periodic vibrations rather than a direct recording of each vibratory cycle. Its interpretation may be affected by examination technique, phonatory task, image quality, irregular vibration, patient cooperation, and examiner experience [6,119].

Supraglottic hyperfunction also requires careful interpretation. This may represent an adaptive response to glottic insufficiency, a coexisting functional disorder, or a factor obscuring the underlying vocal fold configuration. Its presence should not automatically be classified as either an intrinsic feature of presbylarynx or an independent diagnosis without considering the broader phonatory pattern [78,79,116,117].

Even high-quality VLS may fail to identify subtle epithelial or subepithelial abnormalities. Comparative studies have shown that selected lesions, including sulcus vocalis, mucosal bridges, and other subtle mucosal changes, may become evident only during operative inspection and palpation [118]. This does not justify routine suspension microlaryngoscopy in patients with suspected presbylarynx; rather, it supports further investigation when symptoms, functional findings, or clinical suspicion remain discordant with office-based examination findings.

### 8.3. Patient-Reported Outcome Measures (PROMs)

PROMs provide information on how voice disorders affect communication, participation, emotional well-being, and daily life. Their inclusion is particularly important in older adults because the clinical impact of similar presbylaryngeal findings may vary substantially according to communication demands, compensatory capacity, social context, and patient expectations [16,20,80,120,127]. PROMs should complement, not replace, laryngeal and functional assessment.

The Voice Handicap Index (VHI) and its abbreviated version, the VHI-10, are widely used to quantify voice-related functional, physical, and emotional burdens. The VHI-10 provides a brief validated measure suitable for clinical screening and longitudinal follow-up while reducing completion burden [128]. An elevated score indicates a patient-perceived impact but does not identify the anatomical or physiological cause of dysphonia.

The Aging Voice Index (AVI) was developed specifically for older adults and includes concerns relevant to the aging voice, such as reduced projection, increased effort, diminished endurance, and restricted communication participation. The instrument has demonstrated validity and reliability, and subsequent linguistic validation supports its use across different populations [127,129].

The choice of PROM should reflect the clinical purpose, population, available validated language versions, and feasibility of repeated administration. The routine use of multiple overlapping questionnaires is not always necessary and may increase patient burden. When broader psychosocial or participation concerns are present, additional targeted measures should be considered.

PROM scores may not correspond closely to acoustic measurements or the apparent severity of laryngeal morphology [16,20,74,120,130]. Such discordance is clinically informative rather than inherently problematic. Substantial perceived limitations despite mild morphological findings may reflect high communication demands, reduced physiological reserve, hearing difficulty, or limited compensatory capacity. Conversely, pronounced atrophy or bowing with few reported handicaps may support observation rather than active intervention. The serial use of the same validated PROM can also help determine whether treatment produces an improvement that the patient considers meaningful.

### 8.4. Multidimensional Voice Assessment

Functional voice assessment should integrate auditory-perceptual, acoustic, aerodynamic, and patient-reported information using standardized tasks and recording conditions whenever possible [17]. Each domain characterizes a different aspect of voice production, and none are sufficiently specific to diagnose presbyphonia or determine the clinical relevance of presbylaryngeal morphology independently.

Validated rating approaches, including GRBAS (Grade, Roughness, Breathiness, Asthenia, and Strain), may improve consistency and facilitate comparison across examinations [17]. Sustained phonation should be supplemented with connected speech because vocal limitations may become more apparent during continuous or demanding communication tasks.

In acoustic assessment, age-related normative variation is considerable, and individual measures may be influenced by sex, task, recording conditions, vocal intensity, and analysis method [71,120]. No single acoustic threshold can distinguish clinically meaningful presbyphonia from asymptomatic vocal aging. Acoustic measures are most informative when interpreted as part of a cross-domain pattern or used longitudinally under standardized conditions.

Aerodynamic findings are not specific to presbylarynx because they may also be influenced by respiratory disease, reduced respiratory muscle strength, task performance, and patient effort [19,77,82]. When available, aerodynamic data should therefore be interpreted in relation to both glottic configuration and respiratory capacity.

Multidimensional assessments are most informative when the findings converge. For example, bilateral vocal fold bowing accompanied by a glottic gap, breathy hypophonia, reduced phonation time, elevated airflow, and patient-reported limitations provide stronger support for clinically relevant glottic insufficiency than any finding alone. When morphology, functional measures, and symptom burden are discordant, clinicians should reconsider compensatory behavior, communication demands, testing conditions, respiratory reserve, neurological disease, hearing status, and other systemic contributors.

Treatment response may provide additional functional information, but should not be used retrospectively as proof that presbylaryngeal morphology was the sole cause of dysphonia. Improvements may result from enhanced respiratory–phonatory coordination, reduced compensatory tension, improved glottic closure, or changes in vocal behavior without alteration of the underlying structural phenotype [131]. Table 3 summarizes the principal domains of the multidimensional assessment and their contributions to clinical interpretation and decision-making.

### 8.5. Clinical Decision-Making in Older Adults

Clinical decision-making should be guided primarily by symptom burden, functional communication, patient goals, communication demands, physiological reserve, modifiable contributors, and the likelihood that a proposed intervention will address the dominant source of impairment. Because the relationship between presbylaryngeal morphology, perceived handicap, and treatment response is imperfect, morphological severity alone is neither an indication for treatment nor a reliable predictor of therapeutic benefit [16,80,98,117].

Observation and conservative management: Observation is appropriate when presbylaryngeal morphology is identified without clinically meaningful symptoms, communication remains satisfactory, and no suspicious or progressive features are present. Conservative management may include reassurance, education regarding age-related voice changes, hydration and voice use strategies, optimization of modifiable medical factors, and periodic reassessment. Follow-up should be individualized according to symptom trajectory, diagnostic uncertainty, relevant risk factors, communication demands, and patient preference [11,16,80,120].

When to intervene: Active management should be considered when dysphonia meaningfully limits daily communication, occupational performance, social participation, emotional well-being or quality of life. The decision to intervene should reflect concordance among symptom burden, patient-reported outcomes, functional voice assessment, and laryngeal findings, rather than endoscopic appearance alone. Alternative or coexisting treatment targets should be reconsidered before intervention (Section 7) [11,80,98,111,116,117,120].

For appropriately selected symptomatic patients, behavioral voice therapy is commonly considered an initial active treatment. It may improve vocal efficiency, respiratory–phonatory coordination, endurance, and maladaptive compensation while also providing information about the patient’s capacity to benefit from functional intervention [98,117,131].

Procedural and surgical decision-making: Persistent, function-limiting glottic insufficiency despite appropriate conservative management may prompt the consideration of injection augmentation or a more durable medialization procedure. Selection among these approaches should be individualized according to the magnitude and configuration of the glottic gap, expected duration of benefit, reversibility of the intervention, previous treatment response, comorbidity burden, procedural and recovery requirements, and patient priorities [132,133].

Improvement in glottic closure may reduce air loss and phonatory effort but may not fully normalize the voice when respiratory limitation, neurological dysfunction, frailty, cognitive impairment, hearing loss, or maladaptive compensatory behavior remains clinically relevant. These factors should inform patient selection and expectations rather than necessarily precluding intervention [80,98,134]. The subsequent management section discusses specific behavioral, procedural, and surgical treatment options and their supporting evidence.

Shared decision-making and outcome assessment: Shared decision-making should distinguish the anatomical target of treatment from the broader determinants of communication and establish realistic and patient-defined goals. The potential magnitude and duration of the benefits should be considered alongside treatment burden, procedural risk, reversibility, recovery demands, and the patient’s willingness to undergo intervention. Baseline and follow-up evaluations should use comparable perceptual, functional, and patient-reported measures to determine whether treatment produces a meaningful benefit from the patient’s perspective [17,128,131].

A function-oriented paradigm: The central clinical question is not simply whether age-related laryngeal morphology is present, but whether it contributes substantially to the patient’s communication limitations and represents a modifiable therapeutic target. Clinical decision-making should therefore move beyond “What does the larynx look like?” toward “What is limiting this patient’s communication, and which level of intervention is proportionate and likely to help?” [16,80,98,117].

## 9. Treatment Strategies in Presbylarynx

Management should target clinically meaningful voice impairment rather than presbylaryngeal morphology. When dysphonia is functionally significant, treatment planning should integrate symptom burden, patient-reported impact, communication demands, physiological reserve, comorbidity profile, compensatory behavior, and the likelihood that a proposed intervention will address the dominant source of impairment [16,80,133,134,135].

Alternative and coexisting causes should be assessed before attributing dysphonia to presbylaryngeal changes (Section 7). This distinction is clinically important because improving glottic closure may not adequately resolve voice impairment arising predominantly from respiratory, neurological, cognitive, systemic, or compensatory factors [16,80,98,111].

**Figure 6:**
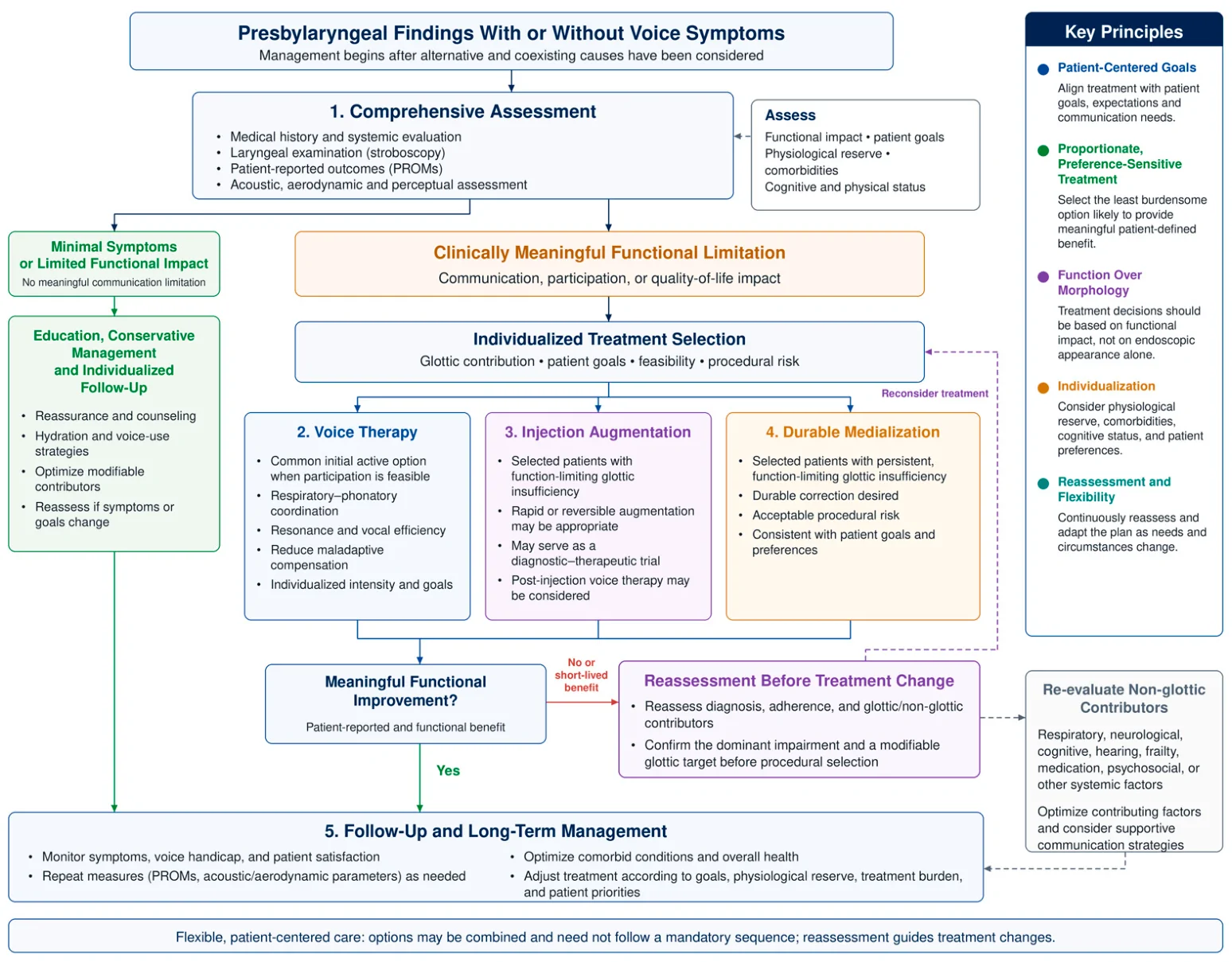
Proposed flexible management pathway for older adults with presbylaryngeal findings. Original schematic created by the authors, integrating assessment principles and treatment evidence [16,111,133,136,137,138]. Selection reflects functional impact, patient goals, and physiological reserve. Inadequate or short-lived benefit prompts reassessment of diagnosis, adherence, and glottic/non-glottic contributors. Options may be combined; the pathway is neither a mandatory escalation sequence nor a validated treatment algorithm. PROMs, patient-reported outcome measures.

Management options include education and observation, behavioral voice therapy, injection augmentation, and durable medialization procedures. These options may be used individually or in combination and should be viewed as components of a flexible clinical framework rather than a mandatory escalation sequence [132,133,134,135]. Current evidence indicates potential benefits from both behavioral and procedural approaches; however, direct comparisons remain limited by heterogeneous diagnostic definitions, small or selected samples, inconsistent outcome measures, and the predominance of nonrandomized intervention studies [132,134,135]. Figure 6 summarizes this flexible, patient-centered management pathway.

### 9.1. Voice Therapy

Indications and therapeutic goals: Behavioral voice therapy is commonly considered the initial active treatment for symptomatic patients who can meaningfully participate in a structured rehabilitation program. It is particularly appropriate when dysphonia is associated with inefficient respiratory–phonatory coordination, reduced vocal intensity or endurance, increased phonatory effort, or compensatory supraglottic hyperfunction [98,117,134,135]. The principal goals are to improve vocal efficiency, audibility, endurance, and communication participation rather than restoring youthful laryngeal anatomy.

Treatment selection should consider cognitive capacity, hearing status, motivation, fatigue tolerance, physical endurance, access to therapy, and the ability to practice and generalize techniques outside clinical settings. Chronological age alone should not determine the patient’s suitability. Conversely, severe frailty, cognitive impairment, or limited capacity for sustained practice may require shorter sessions, simplified exercises, caregiver involvement, or more pragmatic functional goals [98,117,134,135].

Evidence of benefit: A systematic review with meta-analytic synthesis found post-treatment improvements in PROMs, maximum phonation time, and selected acoustic perturbation measures. However, improvement varied across studies, and randomized trials reported more modest improvements in patient-reported outcomes than nonrandomized studies [135].

Prospective studies of vocal function exercises and related approaches have also reported improvements in vocal effort, phonatory efficiency, maximum phonation time, acoustic measures, and voice-related handicap [131,138,139,140].

These findings support voice therapy as an effective option for many appropriately selected patients but do not establish that all individuals with presbylaryngeal morphology will respond similarly to voice therapy. Variability in baseline impairment, treatment content, dose, adherence, outcome selection, and diagnostic criteria contributes to differences in the reported results. Patient-perceived improvement may also occur without substantial change in vocal fold bowing or other static morphological findings, reinforcing the importance of functional rather than anatomical treatment goals [131,135,138,139,140].

Mechanistic considerations: A small prospective electromyographic study reported improvements in thyroarytenoid muscle activity and several functional measures after therapy, suggesting that behavioral intervention may influence neuromuscular recruitment in selected patients with age-related vocal fold atrophy [139]. Given the small sample size and absence of a control group, these findings should be considered preliminary and should not be interpreted as evidence that therapy reverses laryngeal sarcopenia or structural aging.

In the systematic review, interventions predominantly targeting organ functions were associated with larger gains in maximum phonation time than those predominantly targeting skills and habits. This subgroup finding does not establish the superiority of any single therapy protocol [135]. Excessive strengthening demands may be unsuitable when respiratory reserve is limited or maladaptive hyperfunction is prominent [98,117,135].

Treatment intensity and combined approaches: The optimal frequency, intensity, and duration of voice therapy remain uncertain. A small, randomized trial comparing conventional and intensive schedules found improvement with both approaches, without establishing a universally superior dosing strategy [138]. Adherence, feasibility, home practice, and transfer to daily communication may be at least as important as session frequency.

Respiratory muscle training may be considered when reduced respiratory support significantly contributes to voice impairment. Preliminary single-subject evidence suggests that sequential phonation resistance training and inspiratory muscle strength training may improve selected voice outcomes [141]. However, this evidence remains exploratory and insufficient to support the routine use of a standardized combined protocol. Respiratory interventions should be selected based on documented impairment, overall health, and the patient’s capacity to participate safely.

Limitations and reassessment: Limited response to voice therapy should prompt reassessment rather than automatic progression to a procedure. Possible explanations include an inadequate treatment dose, poor adherence, unrecognized neurological or structural disorders, substantial glottic insufficiency, respiratory limitation, or goals that are not adequately addressed by the selected approach. When glottic insufficiency remains a plausible and modifiable contributor, injection augmentation may be considered as a subsequent therapeutic option or, in selected circumstances, as an earlier intervention [98,117,132,133,134,135].

### 9.2. Injection Augmentation

Indications and objectives: Injection augmentation, also referred to as injection laryngoplasty, may be considered when clinically relevant glottic insufficiency is associated with breathiness, reduced vocal intensity, vocal fatigue, increased airflow, or excessive phonatory effort. It is particularly relevant when voice therapy provides insufficient benefit, the glottic gap is substantial, rapid functional improvement is important, or sustained participation in behavioral therapy is not feasible [98,117,133,134].

Appropriate patient selection requires concordance among symptoms, functional voice findings, and observed glottic closure patterns. Vocal fold bowing or atrophy without functionally significant glottic insufficiency is not an indication for injection [98,117,133,134].

Materials, setting, and durability: The available injectable materials differ in terms of viscoelastic properties, resorption profiles, technical requirements, and expected duration of effect. The selection should reflect the intended duration of augmentation, tissue characteristics, previous treatment response, procedural setting, and patient preference. Temporary or intermediate-duration materials may be particularly useful when the expected benefit is uncertain or a reversible intervention is preferred [98,117,133,134].

Office-based injection under local anesthesia may avoid the need for general anesthesia and provide a practical option for selected older adults. However, its suitability depends on patient cooperation, anatomy, airway considerations, anticoagulant or antiplatelet therapy, procedural tolerance, and clinician experience. Advanced age or comorbidity does not automatically make an office-based procedure preferable or risk-free [133,137,142].

Small observational studies have reported improvements in glottic closure, patient-reported handicap, perceptual voice quality, and selected functional measures following injection augmentation in patients with presbyphonia or age-related glottic insufficiency [137,142,143]. Some benefits remain detectable at approximately 12 months in individual cohorts, but durability varies substantially by injectable material, patient characteristics, adjunctive treatment, outcome definition, and threshold for repeat intervention.

A retrospective cohort study reported longer treatment durability among patients receiving hyaluronic acid than among those receiving calcium hydroxyapatite and among patients completing post-injection voice therapy [143]. The study was limited by its sample size, retrospective design, treatment-selection factors, and the use of follow-up or reinjection timing as a measure of durability. Therefore, material-specific superiority and the independent contribution of post-injection therapy require confirmation in prospective comparative studies.

Injection as a therapeutic trial: Temporary augmentation may also function as a reversible therapeutic trial when the contribution of glottic insufficiency to the overall voice disorder is uncertain. Meaningful improvement may support the consideration of repeat augmentation or a more durable medialization procedure. A limited response, however, should not automatically be interpreted as evidence that glottic insufficiency is clinically irrelevant because technical factors, material distribution, insufficient augmentation, compensatory behavior, and non-glottic contributors may all influence the result [98,117,133,137,142,143,144].

Limitations and counseling: Patients should be counseled that augmentation primarily addresses glottic closure and does not reverse age-related changes in the lamina propria, respiratory support, neuromotor control or global physiological reserve. Benefits may be incomplete or temporary, and repeated injections may be required [98,117,133,134].

### 9.3. Medialization Procedures

Indications in selected patients: Bilateral medialization thyroplasty may be considered for persistent, function-limiting glottic insufficiency when durable correction is desired, and less invasive approaches have provided insufficient or unsustained benefits. Suitable candidates generally demonstrate concordant symptoms, functional evidence of inefficient glottic closure, and a laryngeal configuration in which glottic insufficiency appears to be a major contributor to dysphonia [132,133,136].

Surgery should not be regarded as an obligatory final step for all patients who remain symptomatic after voice therapy or injection therapy [98,117,132,133,136].

Evidence and expected outcomes: Retrospective case series have reported improvements in patient-reported and selected objective voice measures following bilateral medialization thyroplasty in patients with presbylarynx [133,136]. However, the evidence base remains limited by small sample sizes, selection bias, heterogeneous techniques and implants, incomplete comparator groups, and variable follow-up. Current data do not establish the superiority of medialization thyroplasty over injection augmentation or behavioral therapy for all patients with presbyphonia [132].

Risk–benefit assessment: Decisions regarding framework surgery should balance the anticipated durability and functional importance of voice improvement with anesthetic considerations, comorbidity burden, physiological reserve, medication exposure, healing capacity, and recovery requirements. Frailty and systemic health should contribute to this assessment, but they should not be treated as absolute contraindications without considering the individual risk profile and expected benefit [16,80,98,117,133,134].

Counseling should clarify that improved glottic closure may reduce air loss and phonatory effort but may not normalize voice when tissue stiffness, respiratory limitation, neurological disease, hearing impairment, or compensatory hyperfunction remain clinically important. Perioperative or postoperative voice therapy may be considered to optimize vocal behavior and respiratory–phonatory coordination, although its incremental benefit following medialization specifically for presbyphonia has not been adequately established [132,133,136].

### 9.4. Conservative Management and Follow-Up

When observation is appropriate: Observation is appropriate when presbylaryngeal morphology is asymptomatic or minimally symptomatic, communication participation remains satisfactory, and no progressive, suspicious, or neurologically concerning features are present. The absence of active treatment should represent a considered clinical decision rather than therapeutic nihilism. Patients should understand that treatment remains available if their symptoms or communication needs change [16,20,80,98,111,116].

Conservative care may include education regarding age-related voice changes, hydration and vocal hygiene strategies, avoidance of harmful voice behaviors, optimization of reflux or inflammatory conditions when clinically established, medication review, hearing support, and management of respiratory or systemic contributors. General physical activity and adequate nutrition may support overall physiological reserve, although they should not be presented as specific treatments for presbylaryngeal morphology [16,80,98,111,116,117].

Follow-up and escalation: The intensity and timing of follow-up should reflect the symptom trajectory, diagnostic certainty, risk factors, communication demands, and patient preference. Reassessment should include changes in voice quality and effort, functional participation, new associated symptoms and patient-defined goals. Repeat laryngeal imaging is appropriate when symptoms progress, new red flags emerge, or the clinical course no longer corresponds to the original diagnosis [80,98,111,116,117].

Treatment escalation should be based on persistent, meaningful functional limitations rather than failure to normalize endoscopic morphology. Combined treatment or earlier procedural intervention may be reasonable when supported by the patient’s impairment pattern and preferences (Figure 6) [98,117,132,133,134,135,136,137,142,143,144].

Use comparable patient-reported, perceptual, acoustic, and aerodynamic measures before and after treatment whenever feasible [17,128,135]. Continued management should be guided by whether the selected intervention improves communication, reduces vocal effort, and meets the patient’s priorities.

## 10. Future Perspectives

Important challenges remain in diagnostic standardization, clinically meaningful phenotyping, prediction of treatment response, and interventions that modify rather than merely compensate for age-related tissue dysfunction [77,134,145,146,147]. Future progress will depend on integrating multidimensional clinical assessment with appropriately validated digital tools, regenerative approaches, advanced biomaterials, and neuromuscular interventions while maintaining patient-centered functional outcomes as the principal therapeutic target.

### 10.1. From Morphology-Based Diagnosis to Multidimensional Phenotyping

Morphological findings do not consistently correspond to the presence or severity of patient-reported voice impairment. Voice outcomes in older adults may also be influenced by respiratory function, frailty, neuromuscular reserve, hearing status, cognition, physical conditioning, medication burden, comorbid disease, compensatory behavior, and individual communication demands [16,18,20,77,80,98].

Future diagnostic frameworks should integrate the complementary assessment domains summarized in Table 3 into multidimensional phenotyping models. Evidence linking frailty with dysphonia and acoustic voice characteristics supports investigating voice within the broader context of biological and functional aging rather than treating it as an exclusively local laryngeal phenomenon [18,148,149].

Such models may ultimately help distinguish patients whose presbylaryngeal morphology remains functionally compensated from those in whom reduced physiological reserve, systemic disease, or increasing communication demands contribute to clinically significant impairment. However, “compensated” and “decompensated” presbylarynx should be regarded as proposed research constructs rather than validated diagnostic categories. Prospective studies are needed to establish reproducible phenotypes, determine clinically meaningful thresholds, and assess whether these classifications predict treatment responses or longitudinal outcomes.

### 10.2. Artificial Intelligence and Digital Voice Biomarkers

Advances in artificial intelligence (AI), machine learning, and computational voice analysis have created opportunities for more objective and scalable assessments of voice and speech. Automated analysis of acoustic recordings may eventually complement clinician-based evaluation, reduce some forms of observer-dependent variability, and support longitudinal monitoring [150,151]. However, the available evidence primarily concerns general voice-disorder classification, computational voice-analysis tools, and voice biomarkers investigated outside specifically characterized presbylarynx populations. Therefore, it does not establish the diagnostic validity of AI for presbylarynx or presbyphonia.

Emerging studies in older adults have identified associations between acoustic voice features and frailty status or frailty phenotypes, and preliminary machine-learning models have demonstrated the feasibility of classifying frailty from recorded speech [148,149,152]. Nevertheless, these studies are predominantly cross-sectional, include relatively small or geographically restricted samples, and were not designed to diagnose presbylarynx or determine the cause of dysphonia. Their findings should not be interpreted as evidence that vocal biomarkers can replace comprehensive geriatric, laryngeal, or functional voice assessments.

Future research should validate AI-assisted tools specifically in well-characterized aging-voice populations, and determine whether they can distinguish presbylaryngeal voice impairment from the neurological, inflammatory, structural, respiratory, and functional causes of dysphonia. External validation across languages, sexes, and age groups, recording devices, acoustic environments, and comorbidity profiles is essential. Attention must also be paid to algorithmic bias, data privacy, interpretability, clinically meaningful performance thresholds, and integration into existing care pathways. The most appropriate role of these technologies is likely to be augmentation of expert clinical judgment and longitudinal follow-up rather than autonomous diagnosis [148,149,150,151,152].

### 10.3. Expanding the Role of Respiratory Assessment

Respiratory aging may modify the functional consequences of presbylaryngeal changes, as discussed in Section 6.2 [77].

Future clinical studies should incorporate objective measures of respiratory reserve, speech-breathing coordination, and respiratory muscle performance, alongside conventional voice assessment. This may improve phenotypic characterization and help identify patients in whom respiratory rehabilitation should complement behavioral or procedural voice treatment. Preliminary evidence suggests that combining phonatory exercise with inspiratory muscle strength training may improve selected outcomes, but the available evidence remains exploratory and does not establish a standardized treatment protocol [141]. Controlled studies are required to identify appropriate candidates, define training intensity, evaluate safety and adherence, and determine whether respiratory interventions provide benefits beyond those of conventional voice therapy.

### 10.4. Regenerative Medicine and Biological Restoration

Current behavioral and medialization-based treatments primarily improve the functional consequences of the presbylarynx without reversing the biological processes underlying age-related tissue degeneration. Approaches under evaluation include platelet-rich plasma (PRP), basic fibroblast growth factor (bFGF; trafermin), stem cell-based interventions, extracellular matrix modulation, and tissue-engineering strategies [145,153,154,155,156,157,158,159].

PRP is of interest because it is autologous and contains biological mediators that can influence tissue repair. Early clinical reports, including retrospective studies involving patients with vocal fold atrophy, presbylarynx, or vocal fold scarring, have described improvements in patient-reported outcomes, voice measures, or mucosal-wave characteristics after PRP injection [145,157]. However, the evidence remains preliminary because of heterogeneous patient populations, limited sample sizes, short or variable follow-up, absence of robust comparator groups, and variability in PRP preparation and injection protocols. Therefore, PRP should be described as an investigational biological intervention rather than an established regenerative treatment for the presbylarynx.

Intracordal basic fibroblast growth factor (bFGF; trafermin) injection has produced encouraging functional and patient-reported outcomes in studies of age-related vocal fold atrophy. In a retrospective cohort, improvements were reported for approximately one year following a single high-dose injection [153]. A separate retrospective propensity score-matched study found greater improvement in voice handicap following trafermin injection than after voice therapy [154]. However, differences in treatment selection, follow-up timing, and residual confounding limit causal inference. Optimal dosing, long-term safety, and durability across populations require further prospective evaluation. Regulatory approval and clinical availability should also be considered when assessing the applicability of this approach across healthcare settings.

Stem cell and tissue-homeostasis studies have expanded understanding of vocal fold regeneration and have identified cell populations and signaling pathways potentially involved in maintaining or restoring vocal fold tissue [155,156,158,159]. Much of this evidence is derived from laboratory, transcriptomic, injury-model, or preclinical research rather than clinical studies of naturally occurring presbylarynx. Consequently, translation will require clarification of the cell source, delivery method, survival and integration, functional tissue organization, oncological safety, immune response, and long-term effects.

### 10.5. Biomaterials and Next-Generation Injectable Therapies

Injectable materials are increasingly being designed not only as passive volume-augmentation agents but also as platforms that support cell integration, extracellular matrix organization, and controlled delivery of biological signals. Experimental hydrogels, extracellular matrix-mimetic scaffolds, and other bioengineered injectable materials aim to combine correction of glottic insufficiency with restoration of tissue properties [146,160].

Preclinical studies indicate that some next-generation hydrogels can provide potentially favorable viscoelastic characteristics and support regenerative processes [146,160]. However, successful translation to aging human vocal folds will require more than just demonstrating increased volume or cellular viability. Candidate materials must reproduce the specialized biomechanical properties necessary for vocal fold vibration while demonstrating biocompatibility, injection precision, predictable degradation, durable functional benefits, and acceptable inflammatory and fibrotic responses. Standardized animal models, long-term safety studies, manufacturing consistency, and well-designed early-phase clinical trials are necessary before these materials can be considered alternatives to established augmentation agents.

### 10.6. Neuromuscular Restoration and Functional Electrical Stimulation

Functional electrical stimulation (FES) has been proposed as a strategy to address intrinsic laryngeal muscle dysfunction and age-related thyroarytenoid atrophy. Ex vivo and animal-model studies have reported changes in muscle volume, glottic closure, vocal efficiency, or tissue mechanical behavior following stimulation protocols [147,161].

However, existing evidence remains preclinical and does not establish clinical efficacy or safety in older adults with presbylarynx. Major unresolved issues include optimal stimulation parameters, electrode placement, treatment duration, durability after stimulation discontinuation, patient tolerability, and the possibility of maladaptive muscle recruitment or tissue injury. Future human studies should prioritize feasibility and safety before evaluating comparative effectiveness.

### 10.7. Toward Precision Laryngology

Collectively, these developments support the movement toward a precision-based model in which treatment selection is informed by the dominant mechanism of impairment rather than by chronological age or laryngeal morphology alone [16,18,20,77,80,98].

Achieving this goal will require consensus definitions of presbylarynx and presbyphonia, standardized reporting of endoscopic findings, validated multidimensional phenotypes, and a core set of comparable patient-reported, perceptual, acoustic, aerodynamic, respiratory, and participation-based outcomes. Prospective multicenter cohorts are needed to characterize the natural history of presbylaryngeal findings and to identify predictors of clinically meaningful deterioration. Comparative trials should determine which patients benefit most from observation, voice therapy, respiratory intervention, injection augmentation, medialization, or combined treatment and evaluate durability, adverse effects, feasibility, cost, and patient-defined benefits.

Future research should also include older adults with frailty and multimorbidity rather than restricting enrollment to unusually healthy or highly selected participants.

The most realistic near-term advance is not replacing established clinical assessment with a single biomarker or technology but integrating complementary information into more accurate, individualized decision-making. Until stronger comparative and longitudinal evidence supports these approaches, their use and interpretation should remain proportional to the respective levels of clinical, translational, or preclinical evidence.

## 11. Conclusions

The presbylarynx represents the morphological expression of laryngeal aging; however, anatomy alone cannot determine its clinical relevance. Age-related changes may provide a structural substrate for voice impairment; however, their functional significance emerges only when interpreted in relation to compensatory capacity, systemic health, physiological reserve, and individual communication demands.

The presbylarynx should not be regarded as inherently pathological or as an indication for treatment. Age-related laryngeal changes may occur in individuals who remain functionally compensated and experience no clinically meaningful vocal complaints. Substantial interindividual variability exists, and similar laryngoscopic findings may be associated with markedly different levels of vocal performance, communication participation, and voice-related quality of life.

Some older adults may also underreport gradually progressive voice impairment or attribute communication difficulties to “normal aging,” potentially delaying clinical evaluation. Therefore, assessment should consider not only the presence of voice complaints but also changes in vocal effort, endurance, audibility, communication participation, and social engagement.

Clinical decision-making should be individualized, system-aware, and function-driven. Clinicians should determine whether presbylaryngeal findings contribute to the patient’s functional impairment while excluding alternative and coexisting causes of dysphonia. Consider treatment when voice changes produce meaningful consequences, and tailor it to symptom burden, patient priorities, communication demands, cognitive and physical capacity, physiological reserve, and expected benefits. Observation, behavioral voice therapy, injection augmentation, and durable medialization should be viewed as complementary and potentially combined components of a flexible care pathway, rather than as competing treatments or a mandatory escalation sequence.

Future progress will depend on standardized diagnostic definitions, reproducible reporting of laryngeal morphology, comparable multidimensional outcome measures, and better identification of factors that predict treatment response. The adoption of emerging approaches should remain proportional to the strength of the supporting clinical evidence. Ultimately, management should aim not to eliminate every manifestation of laryngeal aging but to identify when age-related changes become functionally important and to provide personalized, evidence-informed care that preserves communication, participation, independence, and quality of life throughout aging.

Table 4 summarizes the principal clinical concepts and practice implications of this review.

## Figures and Tables

**Table 1:** Conceptual and clinical distinctions between the presbylarynx and presbyphonia.

Feature	Presbylarynx (Structural)	Presbyphonia (Functional)
Definition	Structural and morphological manifestations of laryngeal aging	Clinically relevant perceptual and functional manifestations of the aging voice
Nature	Anatomical/morphological phenotype	Clinical/functional expression
Symptoms	May be absent; morphology may be an incidental finding	Voice symptoms and/or functional limitations are present, although their severity varies
Laryngoscopic findings	May include vocal fold atrophy, bowing, prominence of the vocal processes, and incomplete glottic closure	Presbylaryngeal findings may be present, but their extent does not reliably predict symptom severity
Functional impairment	Not obligatory and cannot be inferred from morphology alone	Clinically relevant impairment is present, but its nature and magnitude vary among individuals
Diagnostic approach	Endoscopic and stroboscopic identification followed by correlation with symptoms, vocal function, and differential diagnosis	Multidimensional assessment integrating patient-reported, perceptual, acoustic, aerodynamic, and laryngoscopic findings while excluding alternative or coexisting causes
Relationship	May provide an anatomical substrate for presbyphonia but may remain clinically silent	Represents the clinically relevant functional expression of the aging voice and is not synonymous with presbylarynx
Treatment indication	Morphology alone does not require treatment	Treatment is considered when symptoms or functional limitations affect communication or quality of life, according to patient goals and the overall clinical context

Presbylarynx denotes age-related laryngeal morphology, whereas presbyphonia denotes clinically relevant voice impairment associated with aging. Morphological findings and functional impairment may not correspond. Morphology alone does not establish the cause of dysphonia or the need for treatment; interpretation requires multidimensional clinical assessment [7,8,11,13,16,20].

**Table 3:** Multidimensional Assessment Framework for the Clinical Evaluation of Presbylarynx.

Assessment Domain	Key Components	Clinical Contribution
Medical History	Symptom onset, duration, progression, and variability; vocal fatigue and effort; communication demands and occupational voice use; comorbidities and medications; neurological screening; red-flag symptoms	Identifies potential etiologies, red flags, and contributing factors; helps establish functional relevance and urgency of further evaluation
Laryngeal Imaging	Flexible or rigid laryngoscopy; videolaryngostroboscopy, when indicated	Characterizes vocal fold morphology, mobility, and glottic closure and, with stroboscopy, vibratory behavior; helps identify coexisting laryngeal pathology
Patient-Reported Outcome Measures (PROMs)	Voice Handicap Index (VHI) or VHI-10; Aging Voice Index (AVI)	Quantifies patient-perceived voice-related limitation and quality-of-life impact; supports shared decision-making and longitudinal outcome monitoring
Perceptual Voice Assessment	Auditory–perceptual evaluation using standardized tasks and validated rating scales (e.g., GRBAS)	Characterizes clinically salient voice qualities and overall severity; complements instrumental measures
Acoustic Assessment	Fundamental frequency, sound pressure level, cepstral peak prominence, harmonics-to-noise ratio, perturbation measures, and connected-speech measures	Provides quantitative characterization of the voice signal and supports standardized longitudinal comparison; findings are not diagnostic in isolation
Aerodynamic Assessment	Maximum phonation time, mean airflow rate, subglottic pressure estimates, and phonatory efficiency	Characterizes glottic efficiency and respiratory–phonatory interaction; supports interpretation of possible glottic insufficiency within the broader clinical context
Systemic and Functional Evaluation	Frailty and physiological reserve; respiratory, cognitive, hearing, and nutritional status; comorbidity and medication burden; physical activity and communication demands	Characterizes physiological reserve, compensatory capacity, and potential modifiers of vocal function; may inform treatment suitability, risk assessment, and rehabilitation planning
Integrated Clinical Interpretation	Synthesis of history, laryngeal findings, PROMs, perceptual, acoustic, aerodynamic, and systemic assessments	Synthesizes cross-domain findings to support diagnostic formulation and individualized management and provides a baseline for monitoring treatment outcomes

No single assessment domain establishes the clinical significance of presbylaryngeal morphology. Integrated findings support differential diagnosis, individualized management, and longitudinal monitoring [17,80,111,127,128]. AVI, Aging Voice Index; GRBAS, Grade, Roughness, Breathiness, Asthenia, and Strain; PROMs, patient-reported outcome measures; VHI, Voice Handicap Index; VHI-10, 10-item Voice Handicap Index.

**Table 4:** Key clinical concepts and practice implications in the presbylarynx.

No.	Key Message
1.	PRESBYLARYNX IS A MORPHOLOGICAL MANIFESTATION OF LARYNGEAL AGING AND IS NOT INHERENTLY PATHOLOGICAL. Age-related laryngeal changes may occur without clinically meaningful voice impairment and do not, by themselves, indicate a need for treatment.
2.	LARYNGEAL MORPHOLOGY DOES NOT CONSISTENTLY PREDICT FUNCTIONAL VOICE IMPAIRMENT. Similar laryngoscopic findings may be associated with substantially different levels of voice impairment and communication limitation.
3.	PRESBYLARYNX AND PRESBYPHONIA ARE RELATED BUT DISTINCT CONCEPTS. Presbylarynx describes the structural phenotype of the aging larynx, whereas presbyphonia refers to its clinically relevant perceptual and functional expression.
4.	DYSPHONIA IN OLDER ADULTS IS FREQUENTLY MULTIFACTORIAL. Laryngeal aging interacts with neuromuscular control, respiratory function, comorbidities, medications, sensory deficits, cognition, and psychosocial factors.
5.	TREATMENT DECISIONS SHOULD BE GUIDED BY FUNCTIONAL IMPACT RATHER THAN MORPHOLOGY ALONE. Treatment should be considered when voice changes cause meaningful communication difficulty, reduced participation, or impaired voice-related quality of life.
6.	MANAGEMENT SHOULD BE INDIVIDUALIZED, PATIENT-CENTERED, AND SYSTEM-AWARE. A multidimensional assessment integrating structural, functional, and systemic factors is essential for accurate diagnosis and optimal management.

Presbylaryngeal findings are not inherently pathological. Their interpretation requires multidimensional assessment of functional impairment, communication participation, compensatory capacity, systemic health, physiological reserve, and patient goals. Treatment should address clinically meaningful functional impairment rather than morphology alone [16,80,98,111].

## Data Availability

No new data were created or analyzed in this study. Data sharing is not applicable to this article.
